# Acute placebo responsiveness predicts longitudinal expectation effects in antidepressant treatment

**DOI:** 10.1038/s41398-026-04070-x

**Published:** 2026-05-13

**Authors:** Eun Jin Shim, Leonie Schmidt, Jonas Rauh, Gregor Leicht, Matthias Gamer, Ulrike Bingel, Christian Büchel, Winfried Rief, Stefanie Brassen

**Affiliations:** 1 Department of Systems Neuroscience, University Medical Centre Hamburg-Eppendorf, Hamburg, Germany; 2 Department of Psychiatry and Psychotherapy, University Medical Centre Hamburg-Eppendorf, Hamburg, Germany; 3 Department of Psychology, University of Würzburg, Würzburg, Germany; 4 Center for Translational Neuro- and Behavioral Sciences, Department of Neurology, University Medicine Essen, Essen, Germany; 5 Department of Clinical Psychology and Psychotherapy, Philipps-University Marburg, Marburg, Germany

**Keywords:** Human behaviour, Depression

## Abstract

Positive treatment expectations are known to enhance antidepressant efficacy. Experimental placebo studies aim to provide mechanistic insights into this association, yet their findings have not been clearly linked to clinical expectation effects within individual patients. In a randomized crossover trial, 61 participants with major depressive disorder (31 women, 30 men) completed an emotion classification task and mood assessments after administration of a saline nasal spray labeled as either oxytocin (sham oxytocin treatment, deceptive placebo) or saline (control). Forty-five participants subsequently underwent antidepressant treatment and were monitored weekly for treatment expectations and depressive symptoms, with follow-ups extending to three months. Using linear mixed-effect models, we examined trajectories of clinical expectation effects and their prediction by experimental placebo-induced changes in emotional processing. Sham oxytocin treatment acutely induced positive expectations, improved mood, and shifted emotional processing toward positivity. Longitudinal modeling revealed that weekly reductions in depressive symptoms were predicted by higher treatment expectations reported the preceding week. These clinical expectation effects were most pronounced in individuals exhibiting strong acute placebo responses in emotional processing. Overall, our findings indicate that positive treatment expectations facilitate critical aspects of emotional processing in depression and that individual responsiveness generalizes across different treatments and time windows. Experimental placebo assessments offer an ecologically valid model for elucidating mechanisms and predictors of clinical expectation effects and highlight expectation sensitivity as a promising target for treatment optimization.

## Introduction

High rates of placebo responses in antidepressant trials have been attributed to positive treatment expectations [1, 2]. Profound effects are seen in subjective mood reports as well as in neural systems involved in emotional processing and emotion regulation [3]. In line with this, recent experimental data in healthy individuals have shown that a sham oxytocin treatment (deceptive placebo) improves mood, biases emotional processing towards positivity [4] and modulates fronto-limbic neural networks involved in emotional regulation [5, 6].

This is intriguing given the high negativity in depression, which is fundamental to the persistence and prognosis of symptoms [7]. Acute antidepressant administration in depressed patients can boost positivity very early, prior to changes in mood and symptoms, which has been discussed as a mechanism of therapeutic action [8]. However, it remains unclear whether positive treatment expectations alone can produce similar beneficial effects on emotional processing in patients. Identifying such an effect could reveal a potential mechanism underlying placebo-related affective improvements and serve as an experimental model for studying predictors of clinical expectation effects.

So far, research on interindividual differences in clinical placebo responses has yielded mixed results. Discussed predictors and moderators mainly include prior treatment experiences, symptom severity, and contextual factors of placebo induction, such as warmth and competence of the therapist and the type of instruction [9–11]. Certain personality and psychological factors, like optimism [12] or suggestibility [13], have been linked to stronger placebo effects. However, meta-analyses on placebo analgesia show no consistent association between specific personality traits and placebo responsiveness, challenging the utility of such factors for identifying placebo “responders” and “non-responders” [14]. Recently, reinforcement learning models were used to describe antidepressant placebo responses as highly dynamic processes maintained by positive feedback loops between expectancies and mood improvement [15, 16], supporting the idea that most individuals are prone to placebo effects under the appropriate conditions [17]. Early identification of these conditions at the individual level is critical for optimizing expectations, and thus improving treatment effects [10].

Here, we assume that individuals’ responsiveness to an experimental expectation induction may reflect the effectiveness of their treatment expectations during actual antidepressant therapy, provided both are assessed under comparable conditions (e.g., within the same depressive episode). To test this, within-subject designs that evaluate expectation effects under both experimental and clinical conditions in the same patients are essential. In the current study, we therefore combined an experimental, cross-over placebo manipulation during an emotion classification task with a subsequent longitudinal observational study in depressed inpatients undergoing antidepressant treatment. We tested several predictions: (i) sham “oxytocin” treatment enhances mood and positivity processing in depressed inpatients; (ii) expectations of antidepressant efficacy predict changes in depressive symptoms throughout inpatient treatment; (iii) individual placebo responsiveness in the experiment predicts clinical expectation-outcome associations.

Our analysis involved psychometric functions of experimental data and a series of linear mixed effects models on longitudinal data. Furthermore, we explored the roles of mood state and prior experience with antidepressant treatment in both experimental and clinical expectation effects.

## Material and methods

### Participants

This combined, prospective crossover-observational trial (https://drks.de/search/en/trial/DRKS00028733) was approved by the German Ethical Review Authority (Ärztekammer Hamburg, PV 7141) and conducted in accordance with the Declaration of Helsinki. The study followed the Consolidated Standards of Reporting Trials (CONSORT) guidelines for randomized clinical trials (the trial protocol is given in Supplement 2).

**Fig. 1:**
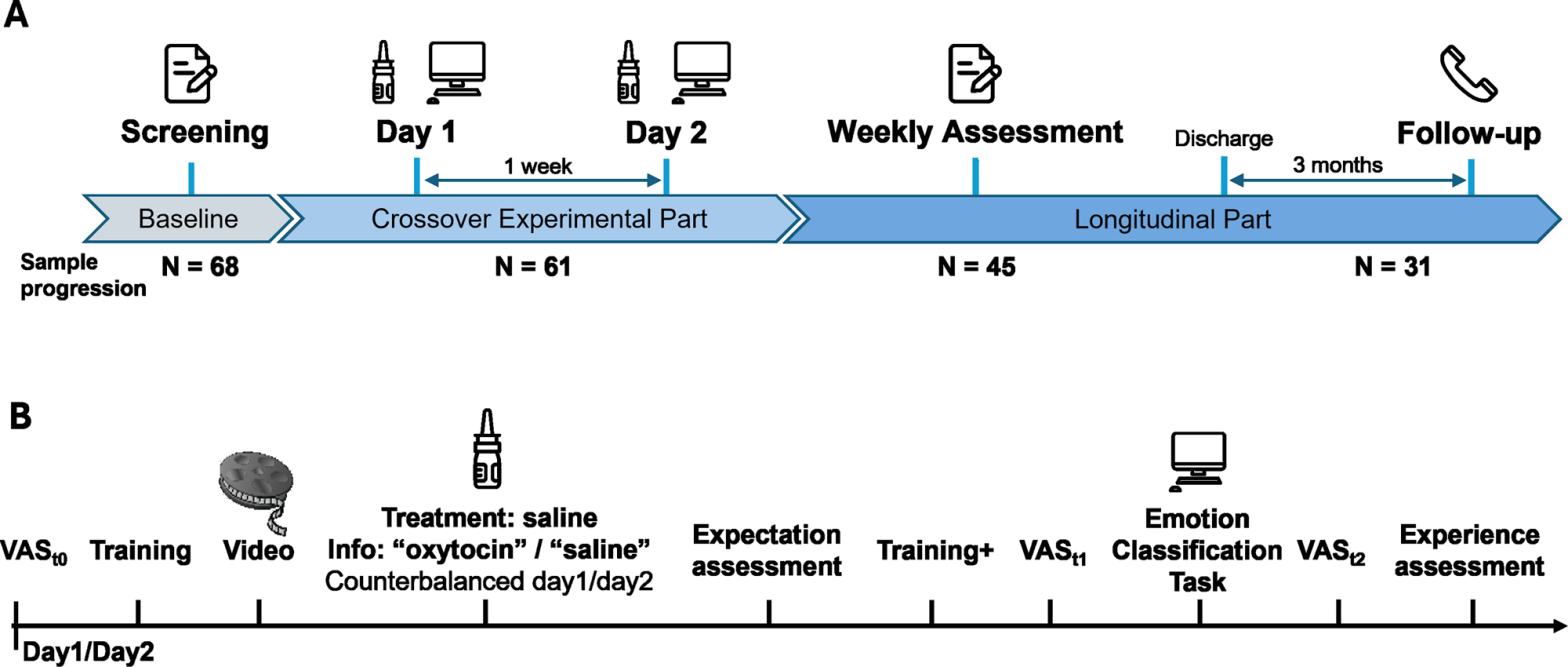
Roadmap and experimental design. **A** Study protocol. **B** Schematic illustration of the experimental design. Each experimental day featured training on the Emotion Classification Task (EC-Task), viewing a standardized oxytocin documentary paired with intranasal self-administration of saline (labeled as either oxytocin or saline in counterbalanced order via random allocation), expectation assessment, the EC-Task itself, and end-of-day ratings of treatment experience. Mood was monitored using visual analogue scales (VAS) at session start (VAS_t0_), pre-EC-Task (VAS_t1_), and post-EC-Task (VAS_t2_) to establish baseline states and detect timing-related effects on experimental outcomes.

Patients diagnosed with acute depressive episodes who were receiving treatment in the psychiatric inpatient unit for depression of the University Medical Centre Hamburg-Eppendorf between June 2022 and July 2023 were recruited. Patients were invited to participate through flyers provided by physicians and announcements during group meetings. Exclusion criteria encompassed neurological diseases, psychotic disorders, substance use disorders, and age over 60. Enrolled participants provided written informed consent and received financial compensation for participation. All participants with ongoing antidepressant treatment were invited to participate in the longitudinal observational study and at a 12-week follow-up assessment (Fig. 1; see also supplementary methods).

### Study design

Participants were not fully informed about all aspects of the study. The deceptive narrative constituted investigating experimental responsiveness to a single-dose oxytocin treatment during emotional processing (crossover part) and its effect on the course of depressive symptoms during the clinical stay (longitudinal part). The screening involved a clinical interview, quantification of depression severity using the Montgomery-Åsberg Depression Rating Scale (MADRS), and a baseline (t0) assessment of our primary outcome for subjective depressive symptoms with the Beck Depression Inventory-II (BDI-II). The cross-over experiment took place on two days separated by approximately one week. Following the second experimental day, enrolled participants of the longitudinal part were assessed weekly until discharge on their depressive symptoms (BDI-II), current antidepressant treatment and related treatment expectations and experiences. The observational assessment was repeated 12 weeks after the experiment via telephone (Fig. 1).

#### Experimental expectation induction

Positive expectations about intranasal oxytocin were induced with a 5-minute video in which an expert explains its mechanisms and mood-enhancing effects. Afterward, participants opened a sealed envelope revealing whether they were in the (alleged) “oxytocin” or “saline” condition. They were told receiving the same substance on both days was possible, with unblinding justified to prevent placebo effects. On the alleged oxytocin day, a hidden behavioral manipulation reinforced the belief that oxytocin enhanced sensitivity to positive emotional cues (see Supplementary Methods for details). In the following, sham oxytocin treatment (saline + oxytocin instruction) is referred to as the (deceptive) placebo condition, while saline + saline instruction is termed the control condition.

#### Emotion classification task

Participants were required to label images of emotional facial expressions of varying intensity presented on an LCD monitor as either happy, fearful, or neutral. Based on previous validation studies [4], images were selected for each emotion that produced 25%, 37.5%, 50%, 62.5%, and 75% correct classifications. Two parallel stimulus sets with 352 images each, matched for difficulty, were counterbalanced across testing days. Each trial started with a fixation cross (0.5 s), followed by the face stimulus (1.5 s), after which participants were required to select “fearful”, “neutral”, or “happy” by button press (see also supplementary methods and Figure S2).

#### Ratings of mood, expectation and experience

Real-time mood state during the experiment was assessed using visual analogue scales (VAS). Treatment expectation and experience were rated on 11-point interval scales of the GEEE (Generic rating scale for previous treatment experiences, treatment expectations, and treatment effects; [18]). Our hypotheses focused on positive prognostic treatment expectations (“How much symptom improvement do you expect from your current antidepressant medication?”) but for the sake of completeness, negative and side-effect expectations and experiences were also assessed in the observational study and included in trajectory and associative models (see also supplementary methods and results).

#### Statistical analyses

Data were processed and analyzed using MATLAB (Mathworks, MA) and R / RStudio (R v4.3.2). Statistical analyses employed the general linear model framework, including repeated measures ANOVA (rmANOVA), one-sample t-tests, Pearson correlation, and multilevel models (LMM). The significance level was set at α = 0.05 (two-sided).

EC-Task data analyses followed previous protocols [4]. Detection accuracy at each intensity and emotion in the control condition served as baseline; responses in the placebo condition were regressed onto control within participants, yielding intercepts and slopes to assess response criterion and sensitivity changes.

Longitudinal data were analyzed with LMMs (lme4), initially with random intercepts and slopes. Model comparisons used likelihood ratio tests, AIC, and BIC; if adding random slopes did not significantly improve fit, results from simpler models with only random intercepts are reported, balancing complexity and fit.

The following models were specified (for detailed parameters and model comparisons see supplement):

##### M1: Trajectory models

To analyze the course of depressive symptoms and antidepressant expectation (dependent variables, DVs) during the treatment period, the predictor time was modelled as both a fixed effect and a random effect to account for individual baseline differences and variability in change across participants (pID):

$${\rm{DV}} \sim {\rm{time}}+(1+{\rm{time}}|{\rm{pID}})$$

##### M2: Associative models

To examine the relationship between depressive symptoms and expectation measures (independent variables, IVs), we tested a model with fixed effects for IV, time, and their interaction, as well as random intercepts and slopes for IV and time:

$${\rm{BDI}} \sim {\rm{IV}}* {\rm{time}}+(1+{\rm{IV}}+{\rm{time|pID}})$$

##### M3: Predictive models

To assess whether prior antidepressant expectations predict changes in subjective depressive symptoms, we created a dependent variable representing BDI change by subtracting weekly scores from baseline scores (BDI(t_0_-t_i_)) und used IVs from the previous week (t_i-1_). Positive BDI(t_0_-t_1_) values hereby denote improvement; thus, β > 0 indicates that IV predicts larger improvements:

$${\rm{BDI}}({{\rm{t}}}_{0}-{{\rm{t}}}_{{\rm{i}}}) \sim {\rm{IV}}({{\rm{t}}}_{{\rm{i}}-1})* {\rm{time}}+(1+{\rm{IV}}({{\rm{t}}}_{{\rm{i}}-1})+{\rm{time|pID}})$$

##### M4: Translational models

Finally, we tested whether potential interactions between BDI change and clinical expectations are related to individual placebo responsiveness in the initial experimental session.

$${\rm{BDI}}({{\rm{t}}}_{0}-{{\rm{t}}}_{{\rm{i}}})* {\rm{IV}}({{\rm{t}}}_{{\rm{i}}-1}) \sim {\rm{IV}}({\rm{experiment}})+(1{\rm{|pID}})$$

## Results

### Placebo effects on expectation, mood state, and post-treatment experience

**Fig. 2:**
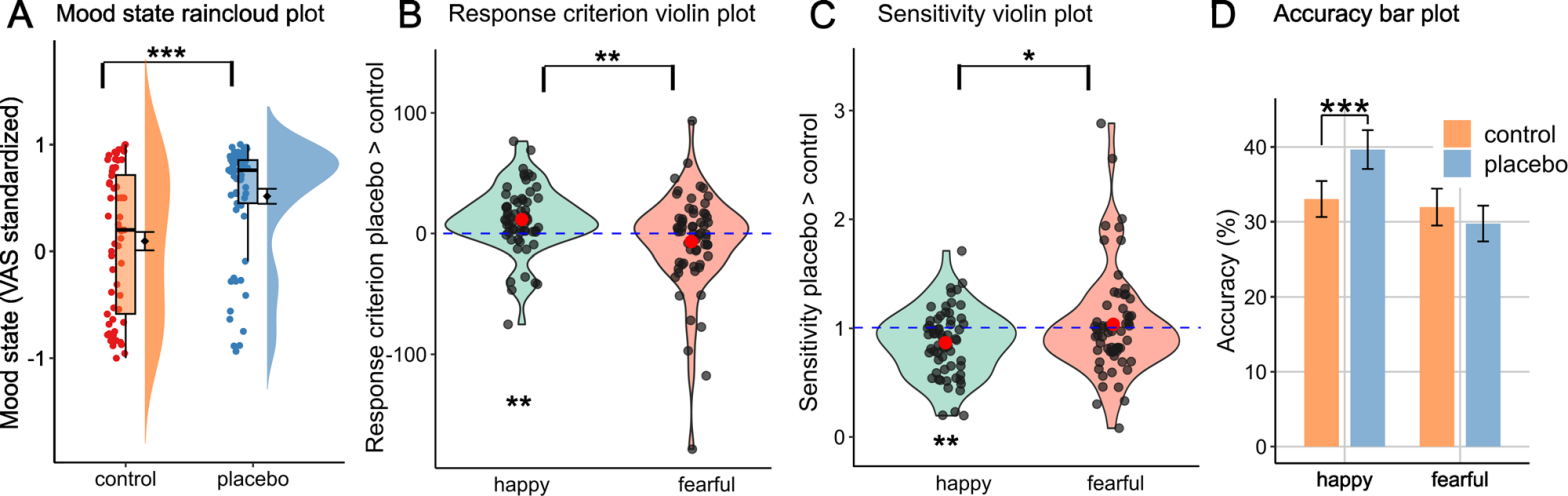
Results of the experimental placebo manipulation. **A** Placebo increased averaged VAS mood ratings, data are range normalized and baseline corrected. Violin plots of individual intercepts (**B**) and slopes (**C**) from psychometric functions of Emotion Classification (EC) Task data. **D** EC-Task accuracy for the lowest 2 steps of expression intensity. ***P < 0.001, **P < 0.01, * *P* < 0.05.

Characteristics of the experimental sample (*N* = 61) are reported in Table 1. Baseline mood state (VAS_t0_) did not differ between sham oxytocin and control session or between day 1 and day 2 (all *P* > 0.83). Similar to our previous studies in healthy individuals [4, 6], participants reported higher positive treatment expectations in the placebo than in the control condition (t_60_ = 14.7, *P* < 0.001, Cohen’s dz = 1.90 [95% CI: 1.47, 2.32]). Baseline- and range-corrected mood ratings were higher following sham oxytocin treatment (F_1, 60_ = 15.25, *P* < 0.001, ηp² = 0.203, Fig. 2A) and this effect increased over the experiment as indicated by a condition x time interaction (F_1, 60_ = 7.35, *P* = 0.009, ηp² = 0.109). Accordingly, recapitulated positive treatment experience was enhanced at the end of the placebo session (t_60_ = 8.34, *P* < 0.001, Cohen’s dz = 1.08 [95% CI: 0.76, 1.39]). Both, changes in real time mood ratings (r_61_ = 0.27 [95% CI: 0.02, 0.49], *P* = 0.035) and post-experiment treatment evaluation (r_61_ = 0.57 [95% CI: 0.37, 0.72], *P* < 0.001) were positively related to changes in expectation ratings following intranasal placebo application.

**Table 1:** Characteristics of the study samples.

	Experimental sample	Observational sample	Follow-up sample
Participants, n	61	45	31
Age, years, mean (SE)	36.27 (1.43)	38.06 (1.61)	37.06 (3.91)
Gender, n (%)			
Female	32 (52.5%)	23 (51.1%)	13 (41.9%)
Male	29 (47.5%)	22 (48.9%)	18 (58.1%)
MADRS score screening, mean (SE)	29.46 (0.94)	29.38 (1.19)	29.16 (1.44)
BDI-II score t0, mean (SE)	32.13 (1.07)	32.33 (1.29)	32.48 (1.70)
Years since first episode, mean (SE)	6.97 (0.95)	7.85 (1.15)	7.95 (1.39)
Primary AD Medication, n (%)			
SSRI	20 (32.8%)	18(40%)	7 (22.58%)
SNRI	11 (18.0%)	9(20%)	7 (22.58%)
NDRI	11 (18.0%)	13(28.89%)	10 (32.26%)
TCA	1 (1.6%)	-	-
TetraCA	4 (6.6%)	3 (6.67%)	3 (4.92%)
AD treatment status, n (%)			
Not under AD	14 (23%)	-	4 (12.9%)
On 1 AD	44 (77%)	42 (93.3%)	25 (80.6%)
On 2 AD	3 (4.9%)	3 (6.7%)	2 (6.5%)
Concomitant medication, n (%)			
Lithium	3 (4.9%)	3 (6.7%)	1 (3.2%)
Atypical antipsychotics	11 (18%)	7 (15.6%)	12 (38.7%)
Benzodiazepines	2 (3.3%)	1 (2.2%)	1 (3.2%)
Thyroid hormone	-	1 (2.2%)	-
Primary diagnosis at admission, n (%)			
Major Depressive Disorder	58 (95.1%)	43 (95.6%)	30 (96.8%)
Bipolar affective disorder, current major depression episode	3 (4.9%)	2 (4.4%)	1 (3.2%)

*SSRIs* selective serotonin reuptake inhibitors, *SNRIs* serotonin and norepinephrine reuptake inhibitors, *NDRIs* norepinephrine-dopamine reuptake inhibitors, *TCAs* tricyclic antidepressants, *TeCAs* tetracyclic antidepressants

### Placebo treatment facilitates the identification of happy facial expressions

In the EC-Task, increased intercept (t_60_ = 3.28, *P* = 0.002, Cohen’s d = 0.83 [95% CI: 0.30, 1.35]) and decreased slope values (t_60_ = −3.36, *P* = 0.001, Cohen’s d = −0.87 [95% CI: −1.39, −0.34]) of psychometric functions indicate that placebo treatment induced a positivity bias by lowering the threshold and biasing face discrimination towards happy facial expressions (Fig. 2B,C). Consequently, accuracy for ambiguous happy faces was increased (Fig. 2D). Effects were specific to happiness detection and thus significantly different to – unchanged – fearfulness detection (all *P* < 0.018). Placebo effects on happy intercepts and VAS mood state were correlated (r_61_ = 0.27 [95% CI: 0.02, 0.49], *P* = 0.046).

### Depression and Treatment Effects on Experimental Results

MADRS depression severity was correlated with the number of neutral faces misclassified as fearful (r_61_ = 0.27 [95% CI: 0.02, 0.49], *P* = 0.034), as well as with reduced discrimination of fearful expressions (r_61_ = −0.28 [95% CI: −0.50, −0.03], *P* = 0.024) in the control condition, indicating a stronger negativity bias in individuals with more severe depression. Depression severity was not associated with the experimental outcomes; however, years since initial antidepressant intake negatively correlated with placebo effects on mood enhancement (r_61_ = −0.25 [95% CI: −0.47, −0.00], *P* = 0.049). During the experiment, *N* = 14 patients were not receiving antidepressants. They did not differ from those under antidepressant treatment (*N* = 47) regarding depression severity and experimental effects on expectation, mood or happy face processing (all *P* > 0.32, see supplementary results).

To further understand the effects of depression on experimental measures, we exploratively compared placebo effects to a previously assessed healthy cohort [4]. The comparisons show that patients misclassified more neutral faces as fearful, but only in the control condition (t_99_ = 2.39, *P* = 0.019, Cohen’s d = 0.48 [95% CI: 0.08, 0.88]), and that they demonstrated stronger placebo effects on positive expectations and treatment experience (all *P* < 0.03). For more details on group comparisons, see supplementary results.

### Multilevel modeling of longitudinal clinical data and their prediction by experimental data

Out of the experimental sample, 45 patients participated in the longitudinal observation study (Table 1).

**Fig. 3:**
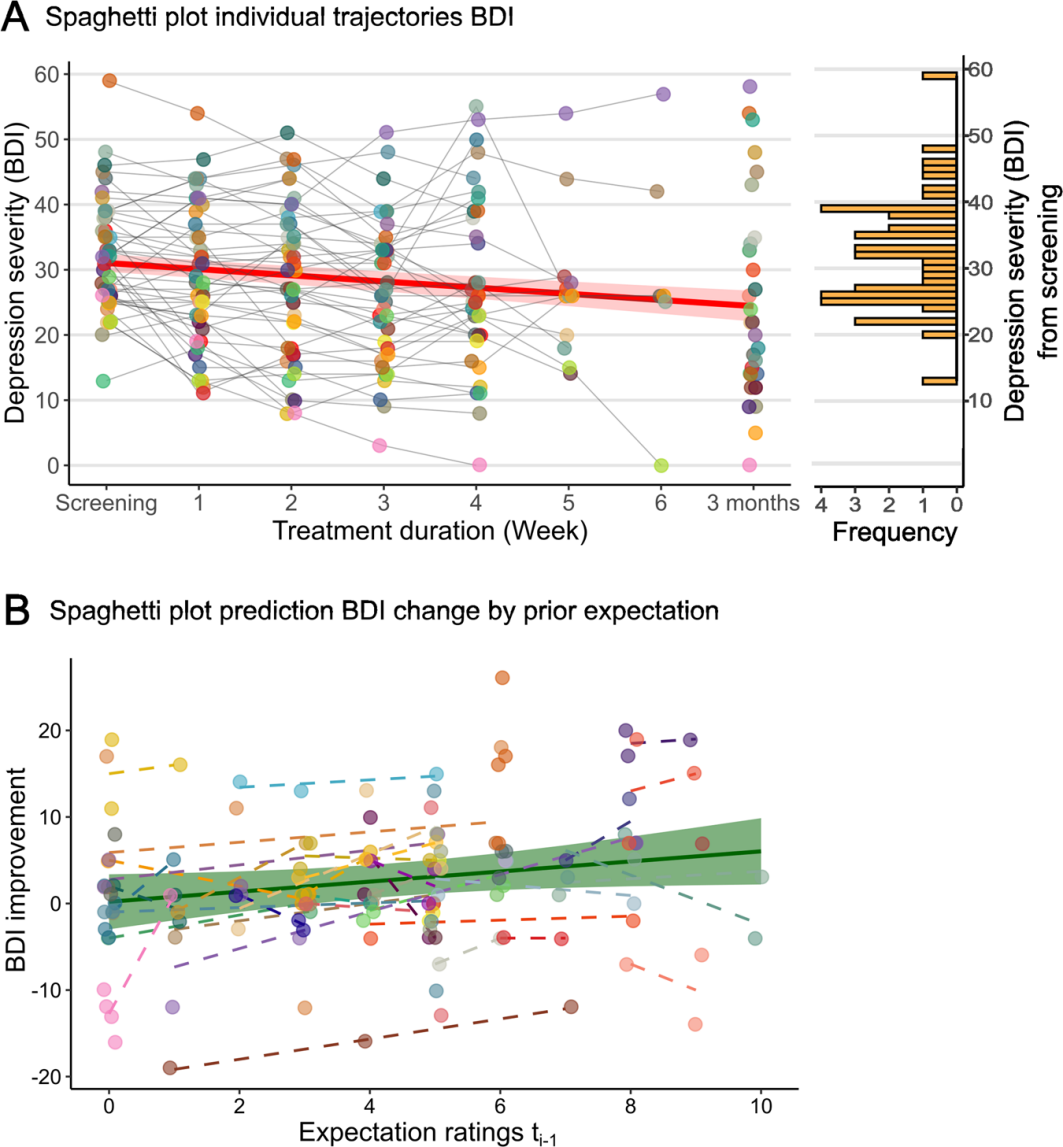
Trajectory and predictive model results. **A** Individual BDI scores from screening to follow-up and model’s regression results across the observational study. **B** Individual and averaged slopes representing the association between the previous week’s positive treatment expectations and BDI change from the first observational assessment (t0). Shaded areas represent 95% CI of model estimates.

Baseline (t0) mean [SE] BDI score was 32.33 [1.29]. Trajectory models demonstrated a linear decrease in depressive symptoms (BDI) over time (ß = −1.50 [95% CI: −2.31, −0.69], *P* < 0.001) with a mean [SE] BDI decrease of 8.84 [2.27] from the first to the last assessment (Fig. 3A). Positive treatment expectations were relatively stable across time (see online supplement for findings on experiences and negative / side effects expectations).

Associative models showed a negative correlation between positive expectations and depressive symptoms (ß = −4.24 [95% CI: −6.24, −2.07], *P* < 0.001). This negative relationship intensified over time, as demonstrated by the interaction between time and expectations (ß = −2.49 [95% CI: −3.71, −1.23], *P* < 0.001).

Results from predictive models extended pure association findings by revealing that weekly reduction in depressive symptoms from baseline (BDI(t_0_-t_i_)) could be predicted by positive antidepressant treatment expectations from the preceding week (t_i-1_) (ß = 2.24 [95% CI: 0.66, 3.82], *P* = 0.007, Fig. 3B).

**Fig. 4:**
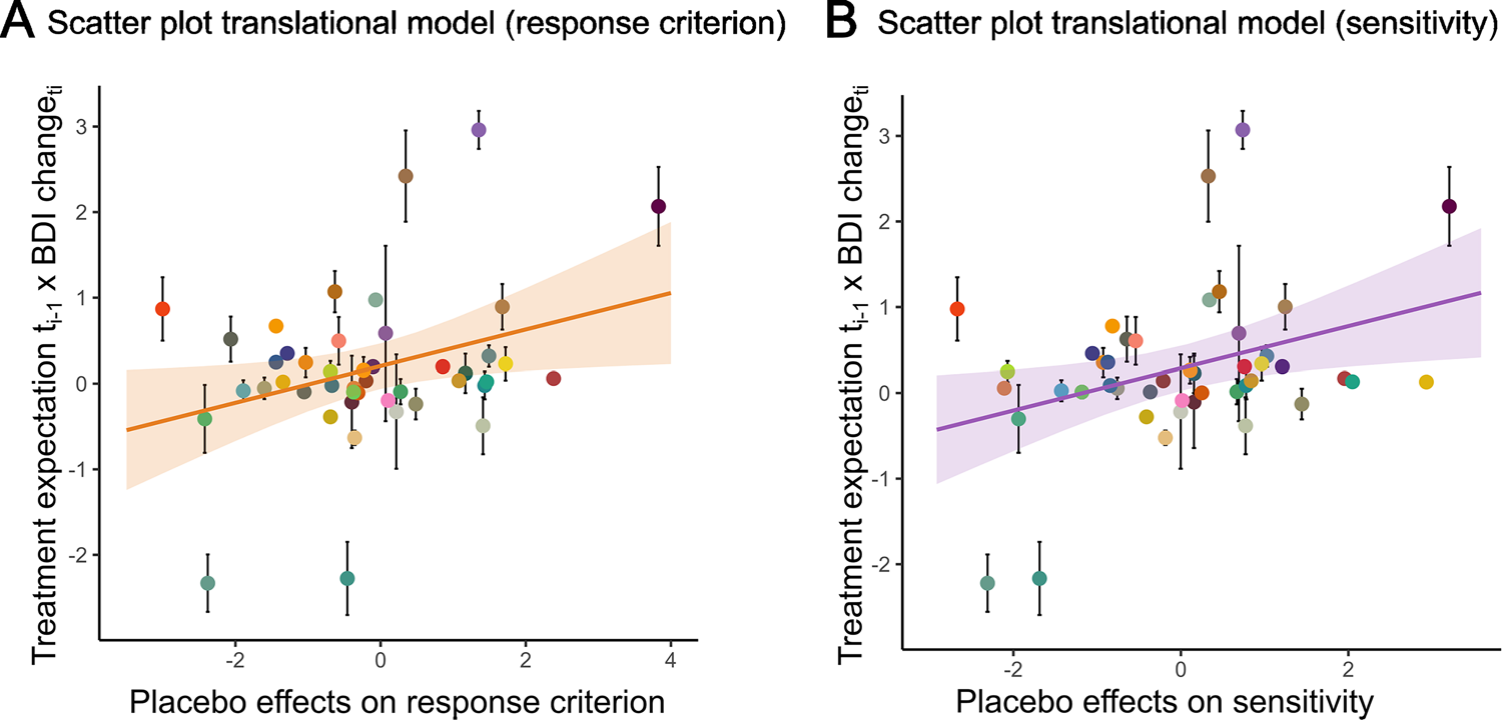
Translational model results. Individual associations between expectations about antidepressant treatment and BDI reduction correlated positively with individual responsiveness to placebo in the emotion classification task (**A**, intercept, **B**, slope, happy > fearful). Error bars represent SE shaded areas represent 95% CI.

Finally, we tested whether the predictive interaction between previous week’s antidepressant expectation and upcoming clinical improvement was more pronounced in patients who had demonstrated stronger positive expectation effects in the initial experiment. Translational models revealed that both indicators of an induced positivity bias in the EC-Task, the intercept (ß = 0.21 [95% CI: 0.02, 0.40], *P* = 0.029) and the slope (ß = 0.25 [95% CI: 0.05, 0.44], *P* = 0.016), were positively related to clinical expectation-outcome predictions (Fig. 4).

### Sensitivity analyses

To verify robustness of our a priori hypothesis-driven results, we conducted post-hoc FDR sensitivity analyses (Benjamini-Hochberg) across experimental (*n* = 7) and longitudinal (*n* = 20) tests. All primary results remain significant post-correction (q ≤ 0.040; Supplemental information, Table S3). Additionally, attrition analyses confirmed robustness against selective dropout. Of the 61 experimental completers, 74% (*N* = 45) entered the longitudinal phase and 51% (*N* = 31) completed the 3-month follow-up. Neither longitudinal nor follow-up completers differed from non-completers in depression severity or experimental effects (all *P* > 0.11). To address potential serial correlation in weekly BDI data, all LMMs were re-fitted with AR(1) residuals, but primary effects remained unchanged (Supplemental Table S6). We further confirmed outlier robustness using non-parametric Wilcoxon tests and robust LMMs (robustlmm package), which showed minimal downweighting and stable results (Supplemental Table S7). Additionally, winsorizing the clinical expectation–outcome measure (5th–95th percentile) in model M4 yielded highly similar estimates (slope β = 0.20, SE = 0.09, *P* = 0.023; intercept β = 0.18, SE = 0.08, *P* = 0.032).

### Follow-up data

Thirty-one participants (69%) from the longitudinal study sample were re-assessed via telephone approximately 12 weeks after the last clinical assessment (Table 1). Mean [SE] BDI was 24.81 [2.74], reflecting a reduction of 11.67 [3.16] points from t0. Including follow-up data in the predictive and translational models shows that findings regarding the predictive role of positive treatment expectations for depressive symptoms and their interaction with experimental responsiveness remain valid even after three months (see online supplement).

### Control for duration and stability of current treatment and depression history

Due to variations in the duration of current treatment and patients’ depression history, we included both parameters as control variables in predictive and translational models. This did not alter the significant results, and neither parameter explained substantial variability in our findings. Similarly, weekly changes in antidepressant treatment had no main or interactive effects on predictive and translational results (see Table S8): Overall, 25 patients (55.6%) experienced at least one antidepressant dose change or switch during the longitudinal observation period, whereas 20 patients (44.4%) did not undergo any antidepressant medication adjustment. Including time-varying, weekly changes (yes / no) as a moderator in our predictive models revealed no corresponding main effects or interactions (all *P* > 0.19), while the expectation effects remained significant (*P* < 0.009). The same held for the translational models linking experimental to clinical data, where dose changes showed no main or interactive effects (all *P* > 0.47), with both expectation-derived parameters retaining significance (slope: β = 0.28, 95% CI [0.07, 0.48], *P* = 0.009; intercept: β = 0.23, 95% CI [0.03, 0.43], *P* = 0.022), Table S8). These results indicate that variations in antidepressant treatment dose did neither influence the clinical prediction of symptom trajectories nor the predictive value of our experimental parameters.

## Discussion

In this combined experimental-observational study, we could identify a potential mechanism underlying antidepressant placebo effects on mood enhancement and provided evidence that individual responsiveness to positive expectations persists across different timescales and treatments. To our knowledge, this is the first study establishing a link between experimentally induced placebo responses on relevant emotional processes and clinical expectation effects within the same depressed patients. Our results highlight the value of cross-sectional placebo manipulations for understanding the mechanisms and predictors of expectation effects for long-term antidepressant treatment outcomes.

In a first step, we could replicate previous findings from studies in healthy individuals showing positive expectation effects induced by sham oxytocin treatment [4, 6]. Notably, depressed patients showed an even stronger effect regarding induced expectations and post-treatment perception when compared post hoc with healthy participants from a previous study [4]. This aligns with prior experimental results [19], possibly reflecting a heightened desire for relief [20], although findings need to be confirmed in future studies including concurrently assessed healthy controls. Unlike previous studies [9], depression severity was not directly related to experimental effects; however longer antidepressant history was negatively associated with mood improvements, consistent with known links between chronicity and poorer placebo responses in clinical trials [21]. We consider our naturalistic design a strength for translational generalizability, as it models real-world conditions where placebo/expectation effects co-occur with ongoing pharmacotherapy. Given the negative association with depression severity, one might even speculate stronger results in treatment-naïve or early-stage patients; our findings nevertheless robustly generalize to a treatment-experienced inpatient population.

The current study helps to understand the common mechanisms behind treatment and placebo effects in depression. Positive expectations fostered a positivity bias in emotion detection, making ambiguous faces more likely to be classified as happy. This can help to overcome negativity biases in depression [22], and to promote social reward sensitivity [23]. Depression severity was related to a negativity bias at baseline in the present study, but this effect might have been underpowered due to the use of fearful instead of more depression-relevant sad faces. This choice was guided by our focus on previously validated effects of this setup on happy faces as the primary outcome [4], which required arousal-matched negative expressions for comparison.

Similar effects on face processing have been observed following single-dose antidepressant treatment in healthy and depressed individuals [8, 24], in medicated versus non-medicated depressed patients [25] and in relation to depressive symptom reduction [26]. While these findings have been interpreted as key elements of antidepressant action, our results indicate that positive treatment expectations alone can induce such modifications. Antidepressant effects on emotional processing have been associated with changes in limbic areas like the amygdala [27], while recent research attributes expectation effects on positivity to prefrontally mediated cognitive control [6]. Thus, while the mechanistic pathways underlying increased positivity may differ between pharmacological antidepressants and placebo effects, learning to respond to a more positive environment likely drives symptom improvement. Importantly, our findings also suggest that educating patients about how treatment can influence emotional processing could significantly enhance the efficacy of active antidepressant therapy.

Responses to antidepressant medication likely involve placebo mechanisms. The exact nature of this combination – whether additive or interactive – remains unclear but the crucial role of positive treatment expectations is well-documented [10, 28–30]. We observed that positive expectations predicted mood improvement during both experimental placebo and active antidepressant treatment. During antidepressant treatment, current prognostic expectations were directly linked to mood and predicted future symptom reductions. This aligns with reinforcement learning findings highlighting the dynamic interplay between expectations, momentary mood, and mood changes in antidepressant placebo responses [16]. Recently, acute placebo responses in resting-state neural networks were predictive of an 8-week antidepressant outcome [15]. Our findings now provide direct evidence that the dynamic association between treatment expectations and clinical improvement over several weeks can be predicted by patients’ acute placebo responses in relevant emotional behavior. These results suggest a shared mechanism of expectation responsiveness across different treatments and timescales.

While our findings indicate that positive treatment expectations make a distinct and temporally preceding contribution to subsequent symptom improvement, these effects should be viewed within the broader context of antidepressant treatment. Symptom change is likely influenced by an interplay of pharmacological, psychological, and contextual factors, including the therapeutic environment and potential engagement in concurrent psychotherapy or behavioral interventions. Although our control analyses showed that changes in antidepressant medication stability did not account for the expectancy–BDI association, other unmeasured or interacting processes may further contribute. Future studies should therefore examine how these factors jointly shape individual trajectories of antidepressant response, for example by modeling additive versus interactive contributions of expectation, medication effects, and ancillary treatments in larger cohorts.

Affective disorders have been linked to over-precise negative prior beliefs [31]. Consequently, treating depression may involve providing the brain with resources to modify its internal model (i.e., priors) of the world to become less pessimistic. Within the predictive coding framework, expectations can be understood as brain states representing prior knowledge about upcoming sensory input, with prefrontally encoded priors guiding the acquisition and interpretation of new information [32, 33]. Predictions may also shape the interpretation of subtle interoceptive cues, such as mood signals [34]. From this theoretical perspective, individual variability in expectation-related symptom improvement observed in our study could reflect differences in how effectively positive priors are integrated, maintained, and updated through coordinated interactions between upstream and downstream neural systems [35]. Future work, combining our behavioral approach with neuroimaging, could examine whether individual profiles of expectation sensitivity can be characterized within such neural systems. These investigations may advance our mechanistic understanding of affective placebo responses and ultimately enable early, individualized interventions that optimize the positive effects of treatment expectations on clinical outcomes.

### Limitations

Our study has several limitations. First, we examined a naturalistic cohort with variability in medication history and treatment duration. While control analyses showed these factors had little impact, larger or more homogeneous samples are needed to clarify their mediating role. Second, our focus was solely on antidepressant treatment; it remains to be tested whether the predictive value extends to non-pharmacological therapies like psychotherapy. Finally, data were collected within a single episode, so the generalizability of expectation effects across episodes awaits confirmation in longitudinal studies.

## Conclusions

In this combined experimental-observational study, we demonstrated that the efficacy of antidepressant treatment relies in part on individuals’ ability to integrate positive treatment expectations in their emotional processing. Findings support the concept of an individual expectation responsiveness that generalizes across different treatments and time windows, at least within the same depressive episode. Beyond elucidating mechanisms of placebo responses in the affective system, our findings may inform clinical research on optimizing antidepressant treatment conditions.

## Supplementary information

Supplemental Material

Supplement 2

## Data Availability

The datasets generated and/or analyzed during the experimental part of the study are available in the Research Box repository (http://researchbox.org/5926). Data of the longitudinal part will be available upon reasonable request to corresponding author.
