# Supplementary material for: Acute placebo responsiveness predicts longitudinal expectation effects in antidepressant treatment
Source: Transl Psychiatry. 2026 May 13;16:241. doi: 10.1038/s41398-026-04070-x (PMC13172539; doi:10.1038/s41398-026-04070-x)
Supplement: Supplementary file 1 — Supplemental Material [file 41398_2026_4070_MOESM1_ESM.docx]

**Supplementary Information**

**Supplementary Methods**

**Figure S1.** Consort flow diagram.

**Figure S2.** Example stimuli and timing of the Emotion Classification Task (EC-Task)

**Supplementary Results**

**Figure S3.** Rating results from the experiment

**Figure S4.** Training data, classification accuracy, and psychometric functions

**Table S1.** Experimental results in depressed and healthy cohorts

**Table S2.** Model comparison – main

**Table S3.** Model results – main

**Table S4.** Model results – experience, negative, and side effect expectations

**Table S5.** Model results – including follow-up

**Figure S5.** Distribution of positive, negative, and side effect expectations

**Table S6.** Model results with autocorrelation diagnostics

**Table S7.** Model results with robustness diagnostics

**Table S8.** Model results for medication stability

**Supplementary Methods**

*Recruitment and participants*

This study recruited 63 participants. Recruitment predominantly targeted patients interested in the experimental component of the study, with the option to also participate in the longitudinal part if receiving ongoing antidepressant treatment. Following enrollment, two participants were excluded: one due to early discharge and another for an undisclosed comorbid psychotic disorder. Thus, the final sample for data analysis consisted of 61 participants (Table 1). Prior to screening, patients had spent an average [SE] of 20.29 [1.99] days in psychiatric care. At screening, mean [SE] MADRS score was 32.13 [1.07].

Following the completion of the experiment, participants were invited for weekly assessments as part of the longitudinal component of the study. Of the initial 61 participants, four declined further participation, and three participated only once, resulting in their exclusion due to insufficient data. Among the remaining 54 participants, nine were no longer on antidepressant medication. Consequently, the final observational sample consisted of 45 participants. The first weekly assessment was conducted on average [SE] 14.69 [0.78] days after screening. Participants completed an average [SE] of 4.00 [0.18] weekly assessments. Thirty-one participants took part in a follow-up by phone, which occurred on average [SE] 92.39 [1.99] days after the end of the experiment (Table S1).

*Medication Status*

As expected after approximately 20 days of inpatient treatment, most patients were under stable antidepressant medication during the experimental phase, with N = 44 (72.1%) receiving antidepressant monotherapy, N = 3 (4.9%) receiving dual antidepressant therapy, and N = 14 (23.0%) not receiving antidepressant treatment. Among those participating in the longitudinal experimental assessments (n = 45), 74.4% did not undergo any change in antidepressant medication between screening and the first longitudinal assessment.

*Power-Calculation*

The calculated sample size for all study components was deemed sufficient to detect at least medium effect sizes (d = 0.50). Assuming an alpha level of 5% and a power of 90%, a minimum of 44 participants was required for the experimental part, which involved repeated measures within factors (G*Power 3.1). For the multilevel models, power analyses conducted using the `simr` package in R, based on 500 simulation runs, indicated that a sample of at least 40 participants would provide a power greater than 90% (93.0%, 95% CI: 90.4%–95.1%) to detect a medium effect size (d = 0.50) on fixed effects, assuming an average of 4 repeated measurements per participant. This suggests that, under the specified conditions, the study is sufficiently powered to identify meaningful longitudinal effects. The initial recruitment strategy accounted for an estimated 30% dropout rate, given the expectation that not all patients would participate in the longitudinal assessments.


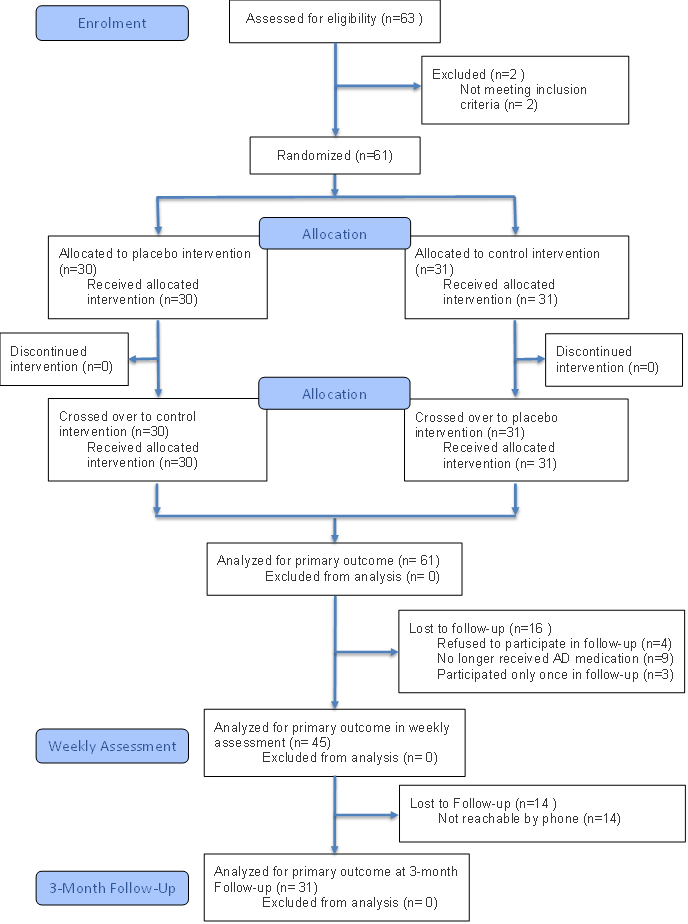


**Figure S1.** Consort flow diagram.

*Emotion Classification Task (EC-Task)*

During both experimental days, participants sat in a comfortable chair with their eyes positioned ~ 100 cm from the center of a 24’’ color LCD screen with a resolution of 1920 x 1080 pixels and a refresh rate of 60 Hz. The task was implemented with MATLAB (Mathworks, US) and the Psychophysics Toolbox extension.

Participants were required to label images of emotional facial expressions of varying intensity as either happy, fearful, or neutral. Stimuli were selected from the Radboud Faces Database (https://rafd. socsci.ru.nl/RaFD2/) and morphed using Abrosoft Fantamorph© to generate a continuum of emotional facial expressions from neutral to happy, and from neutral to fearful, respectively. Based on pilot and validation studies (Baker et al., 2022), two parallel stimulus sets were generated that were matched for difficulty both across sets, and across emotions (Figure S2).

The main task started with a training phase (Training 1) where participants labeled 50 images from 5 identities not included in the experimental sets. After nasal spray administration, a second training phase (Training 2) followed. On the placebo day, happy face intensities in this second training were subtly increased to specifically facilitate happiness classification and thus to reinforce the belief in oxytocin effects (validated in Baker et al., 2022). Following training, participants then completed one of the two task versions, counterbalanced across testing days. Specifically, participants were randomly assigned to conditions and trial sets using a pre-prepared counterbalanced list (created in MATLAB). Trials were pseudo-randomized to avoid consecutive presentations of the same identity. Each set included 352 trials: 8 identities (4 female), showing five intensity levels of happy and fearful expressions plus neutral faces. Each image was shown four times, totaling 32 trials per intensity, 160 per emotion, and 32 neutral trials. Each trial began with a 0.5-second fixation cross, followed by a face for 1.5 seconds (visual angles: 7.72° horizontal, 11.57° vertical). After face offset, response options appeared, prompting participants to categorize the expression as “fearful”, “neutral”, or “happy” by pressing a key. The selected option was highlighted for 165 ms (Figure S2).


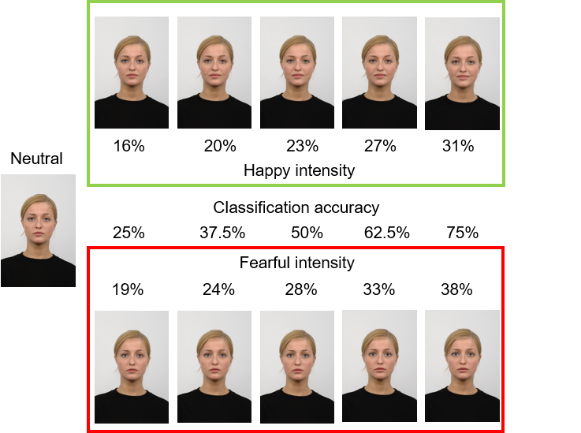

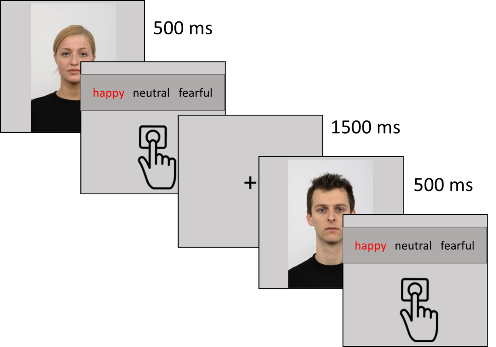


A

B

**Figure S2.** Example stimuli and timing of the Emotion Classification Task (EC-Task). A. Five steps of expression intensity for happy and fearful emotions were used for each identity based on mean accuracy ratings from pilot studies (Baker et al., 2022). Values in the colored boxes represent the approximate expression intensity on the morphing dimension from 0% (neutral) to 100% (happy/fearful). B. Timing of single trials. Faces depicted are used with permission from the Radboud Face Database.

*Ratings*

Participants rated their current mood at the beginning of each testing day (t0), after the second training (VAS_t1), and after completion of the EC-Task (VAS_t2). They were required to move a randomly positioned cursor by using the left or right arrow keys on the keyboard on a continuum that ranged from “unhappy” to “happy” with a resolution of 200 steps. For the analysis, VAS values from each participant of each testing day were scaled relative to the minimum and maximum values to account for individual differences on the range used. Afterwards, values were baseline corrected by subtracting t0 values.

Treatment expectations and experiences were assessed using adapted scales from the Generic rating scale of previous treatment experiences, treatment expectations, and treatment effects (GEEE; Rief et al., 2021). Participants rated on a 11-point scale (0 = no change; 10 = large positive change) their expected and experienced positive mood changes following nasal spray application. Ratings focused on positive effects, as our induction emphasized positive outcomes of oxytocin. Negative and side effects were also explored in the observational study for exploratory proposes, with participants additionally rating expected and experienced mood worsening and side effects from the antidepressant treatment on a 11-point scale.

*Statistical analyses*

**Emotion classification task**

Accuracy values were calculated separately for both placebo and control conditions, as well as for happy and fearful expressions. This involved determining the proportion of trials correctly labeled with the respective emotion at each intensity level, relative to the total number of trials for that level. Detection performance at each step of expression intensity in the control condition served as the baseline. For the placebo condition, performance at each intensity level was regressed onto the control condition performance within each participant, producing a linear regression for each emotion with an intercept and slope. These parameters allowed us to assess changes in response criterion and sensitivity attributable to the placebo manipulation. **Response criterion measurement:** The intercept of the linear regression was used to quantify the response criterion for each emotion. An intercept of zero indicates no change in response threshold due to placebo. A positive intercept signifies a more liberal criterion, i.e., participants were more likely to categorize subtle expressions as the target emotion, while a negative intercept indicates a stricter criterion, requiring clearer signals for labeling. **Discrimination ability measurement:** The slope reflects participants' ability to discriminate subtle emotional expressions. A slope of one means no change in sensitivity under placebo. A slope larger than one indicates increased sensitivity, meaning small differences in emotion intensity lead to larger perceptual differences. Conversely, a slope less than one suggests reduced discrimination sensitivity in the placebo condition compared to control.

**Longitudinal data**

For the analysis of the longitudinal data, we conducted a series of multilevel analyses using linear mixed-effect models (LMMs) with the lme4 package in R (version 4.3.2) within RStudio. Each model targeted specific hypotheses and employed restricted maximum likelihood (REML) estimation, appropriate for datasets with uneven participation across time points, thus maximizing data utilization under the assumption of missing at random (MAR). Models included random intercepts to account for individual baseline differences in the dependent variable and random slopes to model individual variability in response to the predictors. Nested models were compared using the *anova* function from the R *stats* package to assess whether added complexity improved model fit. Model selection was guided by statistical fit criteria, including the Akaike Information Criterion (AIC) and the Bayesian Information Criterion (BIC), with significance tested via likelihood ratio tests. Confidence intervals for model parameters were computed via the *confint* function, providing robust estimates at the 95% confidence intervals. P-values for fixed effects were derived using the *lmerTest* package (version 3.1-3), employing Satterthwaite’s degrees of freedom approximation.

**Supplementary Results**

*Additional results from the experimental study*

At screening, nine patients were receiving antidepressant treatment for the first time. In the remaining 52 patients, the mean [SE] number of prior antidepressant medications was 2.16 [0.47], and the mean [SE] duration since initial intake was 3.77 [0.63] years.

There was no significant difference in baseline mood (t0) between conditions (t_60_ = 0.21, P = 0.83). An rmANOVA on baseline- and range-corrected mood ratings, with factors "condition" (control/placebo) and "time" (t_1_/t_2_), revealed a significant condition effect (F_1, 60_ = 15.25, P < 0.001, ηp² = 0.203), with higher mood ratings in the placebo condition (mean [SE] = 0.52 [0.01]) compared to the control condition (mean [SE] = 0.09 [0.01]). There was no main effect of time. A significant interaction effect between condition and time (F_1, 60_ = 7.35, P = 0.009, ηp² = 0.109) indicated that placebo effects on mood state increased over time (Figure S3, Table S2).


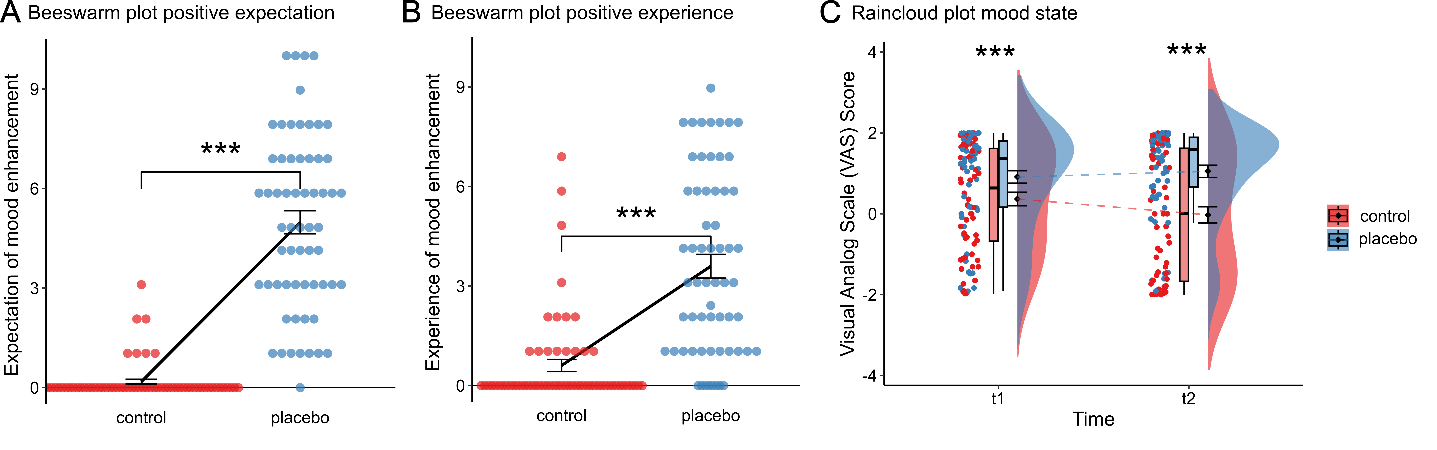


**Figure S3.** Rating results from the experiment. Expectation and experience of mood enhancement through “oxytocin” treatment (A,B). Range- and baseline corrected mood ratings increased under placebo on both time points (C). *** P < 0.001

To further confirm outlier robustness in main experimental effects, we re-ran analyses on placebo effects on mood and happiness detection using non-parametric Wilcoxon tests. Results confirmed that all main effects remained highly significant (placebo effects on VAS mood, expectation, experience, and happy_intercept: all P < 0.001; happy_slope: P = 0.002).

**Training Validation Check**

On the placebo day, the second training (following nasal spray application) used a manipulated stimulus set with slightly increased intensity of happy facial expressions, validated by Baker et al. (2022). As expected, classification accuracy for happy trials was specifically enhanced during this second training on placebo compared to the unaltered control condition (t_60_ = 7.78, P < 0.001, Cohens’ d = 1.00 [95% CI: 0.69, 1.31], Figure S4A/B).


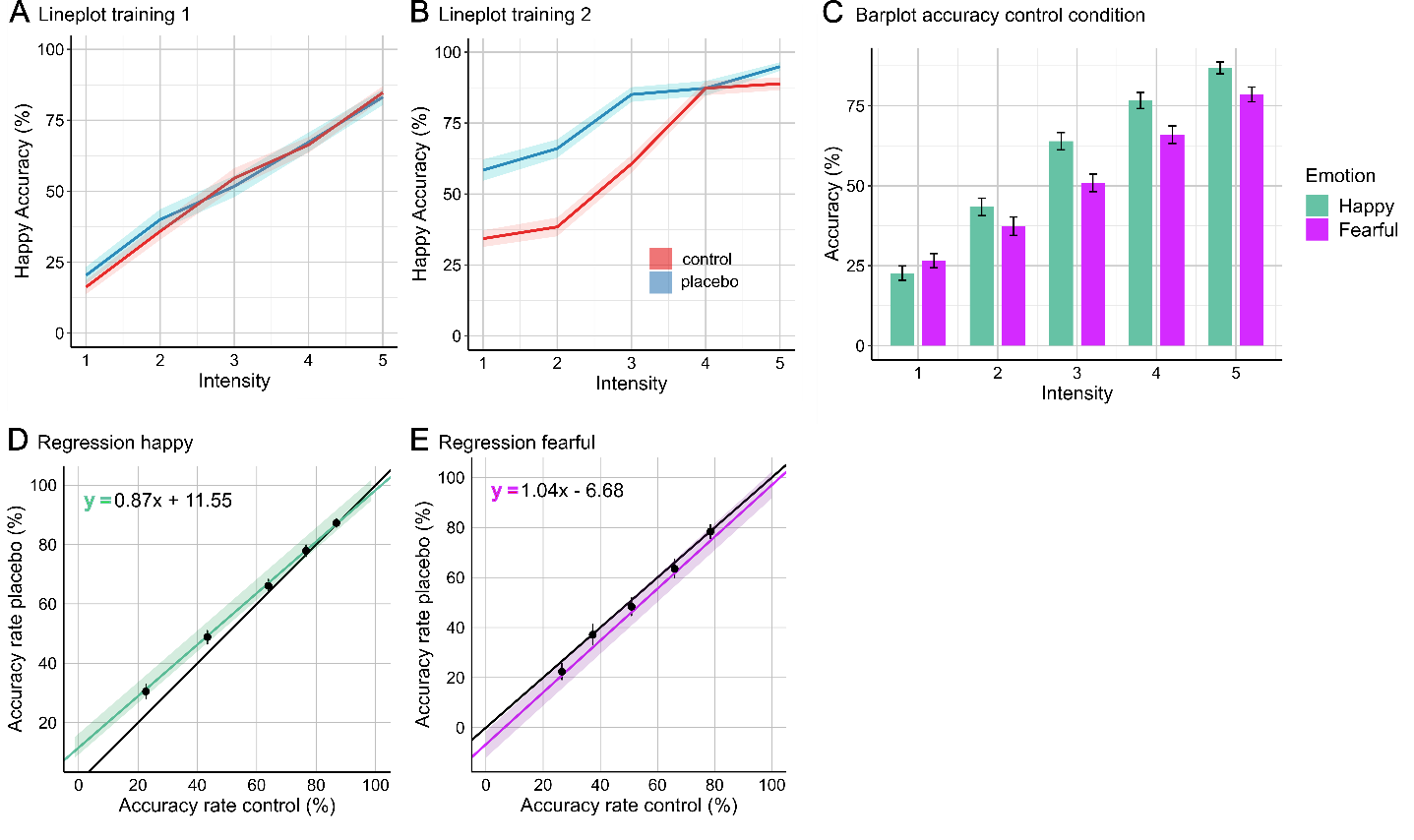


**Figure S4.** Training data, classification accuracy, and psychometric functions. The average classification accuracy along with the standard error of the mean (SE) for happy expressions is shown for the first (A) and the second (B) training set. C. Group averages and SE of classification accuracy during the main task at the control session across five different levels of expression intensity. Mean accuracy rates (SE) were 51% (2.35) for fearful and 58% (2.12) for happy faces. Slightly higher accuracy for happy faces does not reflect a confound since we were interested in within-subject contrasts. D/E. Psychometric functions: Performance in the placebo condition was regressed onto performance in the control condition. Shaded area = SE of intercept; error bars = SE of condition differences at each step of expression intensity.

While this training was intended to enhance participants’ belief in the efficacy of oxytocin treatment, a validation study in N = 12 participants (mean age: 29.7 years, 6 males) using the identical cross-over design but without any treatment application or expectation induction confirmed that the short, manipulated training alone had no effect on subsequent task performance. Specifically, preceding training type had no effect on intercept or slopes for happy (all P > 0.79) or fearful (all P > 0.38) expressions, and accuracy for the lowest two levels of happy expression intensity, which were most affected by placebo in the main study, was likewise unchanged (T_11_ = 1.16, P = 0.27).

**Specificity of results (happy vs. fearful)**

For fearful facial expressions, intercept values did not differ significantly from zero (Figure 2B), and the slope value was not significantly different from 1 (Figure 2C; Table S1). This suggests that the response criterion and detection ability for fearful faces did not change under placebo.

To further assess the specificity of placebo effects on happiness classification, we directly compared effects on happy versus fearful facial expressions by analyzing difference scores of the psychometric functions (intercept and slope, Figure S4). The slope difference between fearful and happy faces was significantly different from zero (t_60_ = 2.44, P = 0.018, Cohens’ d = 0.63 [95% CI: 0.11, 1.15]; Figure 2C). Additionally, the difference in the response criterion (intercept) between happy and fearful faces was significantly greater than zero (t_60_ = 2.99, P = 0.004, d = 0.77 [95% CI: 0.24, 1.29]; Figure 2D), indicating a more liberal response criterion for classifying happy compared to fearful faces (Table S1).

**Order effects**

Although our counterbalanced within-subject crossover design minimizes order effects, we re-ran key analyses with treatment order (placebo first vs. control first) as fixed effect. No order effects emerged for mood or accuracy on subtle happy faces (all F < 0.17, all P > 0.69), while main placebo effects persisted: mood (F_1,59_ = 14.99, P < 0.001, ηp² = 0.203); accuracy (F_1,59_ = 9.62, P = 0.003, ηp²  =  0.14).

**Medication effects**

Participants under antidepressant treatment (n = 47) did not differ from those without antidepressant medication (n =14) regarding treatment expectation ratings (t_59_ = 0.74, P = 0.464). Mood ratings following sham oxytocin likewise showed no group differences (t_59_ = 0.996, P = 0.323). In the emotional classification task, psychometric parameters (slope and intercept) for happy faces also did not differ between groups (all P > 0.371). Similarly, no significant differences were observed in depressive symptom severity at screening, as assessed by MADRS (t_59_ = 0.14, P = 0.888) and BDI-II (t_59_ = −0.22, P = 0.825).

**FDR sensitivity analysis**

FDR sensitivity analysis (Benjamini-Hochberg procedure) across the seven primary experimental variables (mood, expectation, happy slope, happy intercept, fearful slope, fearful intercept) revealed that the originally significant effects (mood, happy slope, happy intercept) remained significant (all P(FDR) < 0.0037) after correction at α = .05, demonstrating robustness against multiple comparison adjustment.

**Belief in oxytocin treatment**

Eighty-eight percent (N = 54) of participants believed they had received oxytocin on the placebo day. Comparing believers and non-believers using Mann-Whitney-U-Test (due to only N = 7 non-believers) showed no differences in placebo effects on mood ratings and EC-Task parameters. However, believers reported significantly stronger effects on experiences of mood enhancement (U = 7.17, P  =  0.007).

**Effects of depression severity**

We investigated whether depression severity measured by the Montgomery-Åsberg Depression Rating Scale (MADRS), was associated with expectation effects and experimental outcomes. Results indicated no significant associations between depression severity and placebo effects on expectation (r_61_ = -0.11, P = .41), experience (r_61_ = -0.07, P = 0.57), mood enhancement assessed via VAS (r_61_ = -0.07, P = 0.59), or placebo effects on happy intercepts (r_61_ = -0.06, P = 0.66) and slopes (r_61_ = 0.09, P = 0.51).

In a post-hoc comparison of the current patient sample to healthy controls from our previous study (Baker et al., 2022), participants with depressive disorders reported higher expectations of mood improvement in the placebo condition compared to control (t_99_ = 2.53, P = 0.013, d = 0.51 [95% CI: 0.11, 0.91]), and greater experiences of mood improvement (t_99.50_ = 2.17, P = 0.025, d = 0.44 [95% CI: 0.04, 0.83]).

Regarding EC-Task parameters, there were no differences between groups in placebo effects on intercepts or slopes for happy faces. For fearful faces, neither response bias nor discrimination ability differed between depressed and healthy participants. Consequently, when directly comparing expectation effects on happy versus fearful faces using difference scores, no group differences were observed (Table S1).

**Table S1.** Experimental results in depressed and healthy cohorts

|  | Depressed cohort (*n* = 61) | | | Healthy cohort (*n* = 40)^4^ | | | Group comparison |
| --- | --- | --- | --- | --- | --- | --- | --- |
|  | Control | Placebo |  | Control | Placebo |  | Placebo vs. control |
|  | *Mean (SE)* | | *P* | *Mean (SE)* | | *P* | *P*³ |
| Expectation | 0.18  (0.07) | 4.98 (0.35) | **< .001^1^** | 1.03  (0.28) | 4.58  (0.37) | **< .001^1^** | **0.013** |
| Experience | 0.61  (0.18) | 3.60 (0.36) | **< .001^1^** | 0.63  (0.24) | 2.48  (0.36) | **< .001^1^** | **0.025** |
| VAS_T0_raw | 176.43 (11.26) | 174.10 (11.09) | 0.832^1^ | 301.20  (10.83) | 311.15  (9.84) | 0.393^1^ | 0.456 |
| VAS_T1_rb | 0.20  (0.09) | 0.48 (0.08) | **0.011^1^** | 0.07  (0.11) | 0.38  (0.12) | **0.050^1^** | 0.838 |
| VAS_T2_rb | -0.01  (0.10) | 0.55 (0.08) | **<.001^1^** | -0.35  (0.13) | 0.01 (0.12) | **0.028^1^** | 0.315 |
| VAS_T12_rb | 0.42 (0.11) | | **< .001²** | 0.32 (0.13) | | **0.023²** | 0.555 |
| Accuracy happy,  step 1+2 | 33.04  (2.41) | 39.65 (2.60) | **0.003^1^** | 33.36  (2.68) | 39.45  (3.07) | **0.046^1^** | 0.885 |
| Accuracy fearful, step1+2 | 31.97  (2.47) | 29.76 (2.39) | 0.469^1^ | 26.95  (2.80) | 27.03  (2.81) | 0.968^1^ | 0.519 |
| Intercept happy | 11.55 (3.52) | | **0.002²** | 12.29 (3.45) | | **< .001²** | 0.887 |
| Intercept fearful | -6.68 (5.30) | | 0.212² | 2.79 (2.88) | | 0.338² | 0.120 |
| Slope happy | 0.87 (0.04) | | **<.001²** | 0.86 (0.05) | | **0.004²** | 0.863 |
| Slope fearful | 1.04 (0.06) | | 0.565² | 0.89 (0.05) | | **0.040²** | 0.106 |
| Intercept happy - fearful | 18.23 (6.08) | | **0.004²** | 9.50 (4.76) | | 0.053² | 0.261 |
| Slope happy - fearful | 0.17 (0.07) | | **0.018²** | 0.04 (0.06) | | 0.582² | 0.185 |

^1^ Paired T-Test. ^2^ One sample T-Test. ^3^ Two sample T-Test. All two-sided. In cases of unequal variances (Levene test), we reported the t-test results that employed the corrected degrees of freedom and adjusted significance values (Welch's t-test). Expectation and experience were assessed using a 11-point Likert scale. VAS = visual analogue scale for mood ratings. raw = raw value. rb = range- and baseline-corrected. VAS_T12_rb = mean VAS change (baseline-corrected and ranged values) on placebo vs. control day. Intercept values = response criterion. Slope values = discrimination ability. ^4^ Data were published in Baker et al. (2022).

*Additional results from the longitudinal part*

The following tables summarize model comparisons, final model parameters, and statistics from all models described in the main manuscript.

**Table S2.** Model comparison – main

|  | AIC | BIC | logLik | Deviance | χ² diff | df | P |
| --- | --- | --- | --- | --- | --- | --- | --- |
| **Trajectory models** |  |  |  |  |  |  |  |
| *BDI ~ time* |  |  |  |  |  |  |  |
| *+ (1 \| pID)* | 1580.1 | 1593.8 | -786.0 | 1572.1 |  |  |  |
| *+ (1 + time \| pID)* | 1538.0 | 1558.5 | -763.0 | 1526.0 | 46.1 | 2 | **<.001** |
|  |  |  |  |  |  |  |  |
| *Positive Expectations ~ time* |  |  |  |  |  |  |  |
| *+ (1 \| pID)* | 760.3 | 773.0 | -376.2 | 752.3 |  |  |  |
| *+ (1 + time \| pID)* | 753.5 | 772.6 | -370.7 | 741.5 | 10.9 | 2 | **0.004** |
|  |  |  |  |  |  |  |  |
| **Associative model** |  |  |  |  |  |  |  |
| *BDI ~ Positive expectations*time* |  |  |  |  |  |  |  |
| *+ (1 \| pID)* | 1179.8 | 1198.2 | -583.9 | 1167.8 |  |  |  |
| *+ (1 + positive expectations \| pID)* | 1175.8 | 1201.2 | -579.9 | 1159.8 | 7.9 | 2 | 0.019 |
| *+ (1 + positive expectations + time \| pID)* | 1157.5 | 1192.3 | -567.8 | 1135.5 | 24.3 | 3 | **< .001** |
|  |  |  |  |  |  |  |  |
| **Predictive model** |  |  |  |  |  |  |  |
| *BDI (t0-ti) ~ positive expectations (ti-1) * time^1^* |  |  |  |  |  |  |  |
| *+ (1 \| pID)* | 894.3 | 911.6 | -441.1 | 882.3 |  |  |  |
| *+ (1 + positive expectations (ti-1) \| pID)* | 898.1 | 921.2 | -441.0 | 882.1 | 0.2 | 2 | 0.909 |

### ^1^The primary goal of the predictive model was to examine how expectations influence changes in depressive symptoms over time. Initially, we specified a model with random slopes for both `time` and `expectation`. However, including a random slope for `time` caused convergence issues, likely due to over-parameterization given our data. To ensure stability, we simplified the model by removing the random slope for `time`, focusing on individual variability in expectations. Despite similar AIC and BIC values and a non-significant model comparison, we also report results of the more complex model because it better reflects our hypothesis that individual differences in expectations are linked to symptom change over time.

**Table S3.** Model results – main

|  | ß | 95% CI | t | P  (orig.) | P  (FDR) | R² (cond.) | R² (marg.) |
| --- | --- | --- | --- | --- | --- | --- | --- |
| **Trajectory models** |  |  |  |  |  |  |  |
| *BDI ~ time + (1 + time \| pID)* | | | | | | | |
| Intercept | 31.80 | 29.03, 34.56 | 22.80 | **<.001** | **0.003** | 0.849 | 0.045 |
| Time | -1.50 | -2.31, -0.69 | -3.68 | **<.001** | **0.003** |  |  |
| *Positive expectations ~ time + (1 + time \| pID)* | | | | | | | |
| Intercept | 4.18 | 3.26, 5.12 | 8.89 | **<.001** | **0.003** | 0.798 | 0.002 |
| Time | 0.09 | -0.13, 0.31 | 0.81 | 0.418 | 0.418 |  |  |
| **Associative model** |  |  |  |  |  |  |  |
| *BDI ~ positive expectations * time + (1 + positive expectations + time \| pID)* | | | | | | | |
| Intercept | 27.61 | 24.33, 30.93 | 16.61 | **<.001** | **0.003** | 0.916 | 0.104 |
| Positive expectations | -4.24 | -6.24, -2.07 | -4.25 | **<.001** | **0.003** |  |  |
| Time | -1.76 | -3.19, -0.30 | -2.40 | **0.017** | **0.024** |  |  |
| Positive expectations x time | -2.49 | -3.71, -1.23 | -4.32 | **<.001** | **0.003** |  |  |
| **Predictive model^1^** |  |  |  |  |  |  |  |
| *BDI (t0-ti) ~ positive expectations (ti-1) * time + (1 + positive expectations (ti-1) \| pID)* | | | | | | | |
| Intercept | 3.79 | 1.71, 5.87 | 3.61 | **<.001** | **0.003** | 0.588 | 0.074 |
| Positive expectations | 2.24 | 0.48, 4.00 | 2.52 | **0.013** | **0.022** |  |  |
| Time | 1.90 | 0.62, 3.17 | 2.93 | **0.004** | **0.009** |  |  |
| Positive expectations x time | 1.13 | -0.13, 2.41 | 1.75 | 0.083 | 0.098 |  |  |
| *BDI (t0-ti) ~ positive expectations (ti-1) * time + (1 \| pID)* | | | | | | | |
| Intercept | 3.77 | 1.66, 5.88 | 3.50 | **<.001** | **0.003** | 0.597 | 0.070 |
| Positive expectations | 2.24 | 0.66, 3.82 | 2.76 | **0.007** | **0.013** |  |  |
| Time | 1.84 | 0.60, 3.08 | 2.89 | **0.005** | **0.010** |  |  |
| Positive expectations x time | 1.17 | -0.06, 2.42 | 1.85 | 0.066 | 0.083 |  |  |
| **Translational models** |  |  |  |  |  |  |  |
| *BDI (t0-ti) * positive expectations (ti-1) ~ response criterion_happy>fearful_ (experiment) + (1 \| pID)* | | | | | | | |
| Intercept | 0.20 | -0.06, 0.46 | 1.52 | 0.131 | 0.138 | 0.527 | 0.069 |
| Response criterion _happy>fearful_ | 0.21 | 0.02, 0.40 | 2.20 | **0.029** | **0.039** |  |  |
| *BDI (t0-ti) * positive expectations (ti-1) ~ sensitivity_happy>fearful_ (experiment) + (1 \| pID)* | | | | | | | |
| Intercept | 0.20 | -0.06, 0.46 | 1.52 | 0.131 | 0.138 | 0.528 | 0.085 |
| Sensitivity _happy>fearful_ | 0.25 | 0.05, 0.44 | 2.45 | **0.016** | **0.024** |  |  |
|  |  |  |  |  |  |  |  |

R² (conditional): Reflects the proportion of variance explained by both fixed and random effects in a mixed-effects model.

R² (marginal): Reflects the proportion of variance explained by only the fixed effects in a mixed-effects model.

P (FDR): P-values corrected for multiple comparisons using the Benjamini-Hochberg false discovery rate (FDR) procedure to control type I error inflation across 20 tests.

**Experiences, negative expectations and side effects**

Trajectory models showed that expectations of mood worsening and positive experiences remained relatively stable over the course of the clinical stay. However, expectations of side effects significantly decreased over time. Associative models revealed no association between the course of depressive symptoms and negative or side effect expectations, but a significant association with positive experiences. Detailed results are provided in Table S4 and Figure S5.

**Table S4.** Model results – experience, negative and side effect expectations

|  | ß | 95% CI | t | P | R² (cond.) | R² (marg.) |
| --- | --- | --- | --- | --- | --- | --- |
| **Trajectory models** |  |  |  |  |  |  |
| *Positive experience ~ time + (1 + time \| pID)* | | | | | | |
| Intercept | 3.25 | 2.38, 4.13 | 7.38 | **<.001** | 0.810 | 0.004 |
| Time | 0.13 | -0.11, 0.36 | 1.08 | 0.282 |  |  |
| *Negative expectations ~ time + (1 + time \| pID)* | | | | | | |
| Intercept | 0.80 | 0.26, 1.33 | 2.97 | **0.003** | 0.418 | 0.001 |
| Time | -0.03 | -0.20, 0.14 | -0.35 | 0.729 |  |  |
| *Side effect expectations ~ time + (1 + time \| pID)* | | | | | | |
| Intercept | 1.97 | 1.38, 2.57 | 6.60 | **<.001** | 0.663 | 0.019 |
| Time | -0.17 | -0.31, -0.02 | -2.49 | **0.014** |  |  |
| **Associative models** |  |  |  |  |  |  |
| *BDI ~ positive experiences * time + (1 + positive experiences + time \| pID)* | | | | | | |
| Intercept | 27.40 | 24.48, 30.35 | 18.46 | **<.001** | 0.905 | 0.222 |
| Positive experiences | -5.86 | -7.76, -3.91 | -6.06 | **<.001** |  |  |
| Time | -1.47 | -2.66, -0.27 | -2.42 | **0.017** |  |  |
| Positive experiences x time | -1.12 | -2.23, -0.03 | -2.01 | **0.046** |  |  |
| *BDI ~ negative expectations * time + (1 + time \| pID)^1^* | | | | | | |
| Intercept | 26.99 | 23.52, 30.45 | 15.39 | **<.001** | 0.856 | 0.021 |
| Negative expectations | 1.00 | -1.04, 3.05 | 0.95 | 0.342 |  |  |
| Time | -1.92 | -3.45, -0.38 | -2.45 | **0.015** |  |  |
| Negative expectations x time | 1.09 | -0.41, 2.59 | 1.42 | 0.156 |  |  |
| *BDI ~ side effect expectations * time + (1 + time \| pID) ^1^* | | | | | | |
| Intercept | 26.93 | 23.47, 30.38 | 15.39 | **<.001** | 0.854 | 0.020 |
| Side effect expectations | 0.60 | -1.20, 2.44 | 0.65 | 0.515 |  |  |
| Time | -1.97 | -3.47, -0.47 | -2.57 | **0.011** |  |  |
| Side effect expectations x time | 0.74 | -0.69, 2.15 | 1.02 | 0.309 |  |  |
|  |  |  |  |  |  |  |

*^1^* model comparison showed better fit for less complex model


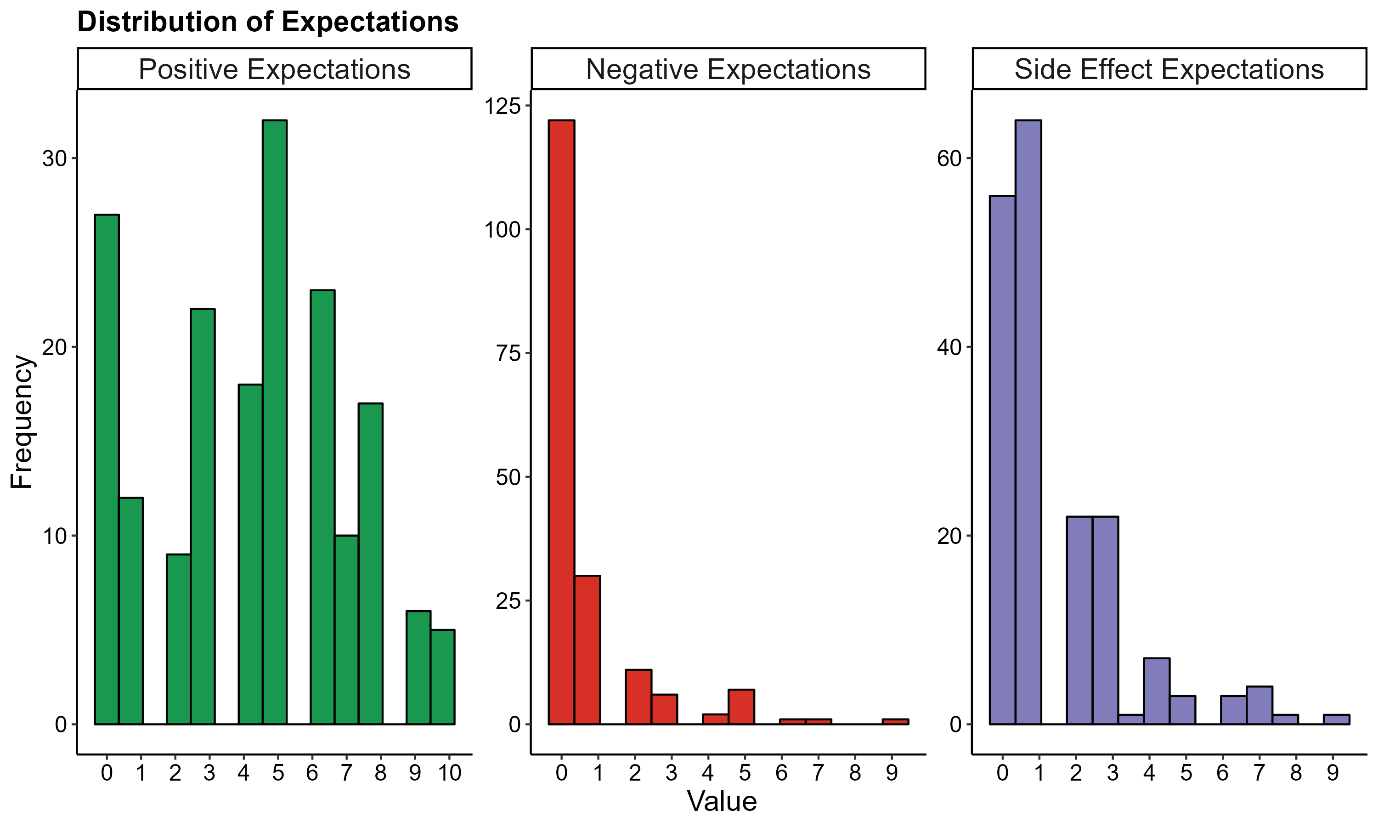


**Figure S5.** Distribution of Positive, Negative and Side Effect Expectation Ratings

**Follow-up results**

LMMs including follow-up data confirm the results observed during the clinical stay.

**Table S5.** Model results – including follow-up

|  | ß | 95% CI | t | P | R² (cond.) | R² (marg.) |
| --- | --- | --- | --- | --- | --- | --- |
| **Trajectory models** |  |  |  |  |  |  |
| *BDI ~ time + (1 + time \| pID)* | | | | | | |
| Intercept | 31.04 | 28.31, 33.77 | 22.53 | **<.001** | 0.824 | 0.030 |
| Time | -0.95 | -1.54, -0.34 | -3.12 | **0.002** |  |  |
| *Positive expectations ~ time + (1 + time \| pID)* | | | | | | |
| Intercept | 4.39 | 3.53, 5.25 | 10.15 | **<.001** | 0.798 | 0.001 |
| Time | -0.01 | -0.17, 0.14 | -0.17 | 0.869 |  |  |
| **Associative model** |  |  |  |  |  |  |
| *BDI ~ positive expectations * time + (1 + positive expectations + time \| pID)* | | | | | | |
| Intercept | 27.59 | 24.05, 30.93 | 15.75 | **<.001** | 0.884 | 0.084 |
| Positive expectations | -3.67 | -5.25, -2.09 | -4.59 | **<.001** |  |  |
| Time | -1.41 | -2.57, -0.26 | -2.42 | **0.017** |  |  |
| Positive expectations x time | -1.27 | -2.28, -0.26 | -2.47 | **0.014** |  |  |
| **Predictive model** |  |  |  |  |  |  |
| *BDI (t0-ti) ~ positive expectations (ti-1) * time + (1 + positive expectations (ti-1) \| pID)* | | | | | | |
| Intercept | 2.63 | 0.72, 4.54 | 2.73 | **0.007** | 0.579 | 0.084 |
| Positive expectations | 1.93 | 0.19, 3.69 | 2.19 | **0.030** |  |  |
| Time | 1.15 | 0.15, 2.16 | 2.27 | **0.025** |  |  |
| Positive expectations x time | 1.12 | 0.12, 2.12 | 2.19 | **0.030** |  |  |
| *BDI (t0-ti) ~ positive expectations (ti-1) * time + (1 \| pID)* | | | | | | |
| Intercept | 2.63 | 0.64, 4.62 | 3.50 | **<.001** | 0.576 | 0.076 |
| Positive expectations | 1.86 | 0.38, 3.34 | 2.76 | **0.007** |  |  |
| Time | 1.09 | 0.11, 2.08 | 2.16 | **0.032** |  |  |
| Positive expectations x time | 1.11 | 0.13, 2.10 | 2.21 | **0.029** |  |  |
| **Translational models** |  |  |  |  |  |  |
| *BDI (t0-ti) * positive expectations (ti-1) ~ response criterion_happy>fearful_ (experiment) + (1 \| pID)* | | | | | | |
| Intercept | 0.16 | -0.08, 0.41 | 1.30 | 0.194 | 0.539 | 0.062 |
| Response criterion _happy>fearful_ | 0.20 | 0.01, 0.39 | 2.07 | **0.040** |  |  |
| *BDI (t0-ti) * positive expectations (ti-1) ~ sensitivity_happy>fearful_ (experiment) + (1 \| pID)* | | | | | | |
| Intercept | 0.16 | -0.08, 0.41 | 1.52 | 0.131 | 0.541 | 0.080 |
| Sensitivity _happy>fearful_ | 0.23 | 0.04, 0.43 | 2.36 | **0.020** |  |  |
|  |  |  |  |  |  |  |

**Table S6.** Model results with autocorrelation diagnostics

|  | ß | 95% CI | t | P | AR(1) ρ^1^ | DW (P)^2^ |
| --- | --- | --- | --- | --- | --- | --- |
| **Trajectory models** |  |  |  |  |  |  |
| *BDI ~ time + (1 + time \| pID)* | | | | | | |
| Intercept | 31.88 | 29.18, 34.57 | 23.33 | **<.001** | 0.32 | 2.03  (0.594) |
| Time | -1.48 | -2.29, -0.67 | -3.61 | **<.001** |  |  |
| *Positive expectations ~ time + (1 + time \| pID)* | | | | | | |
| Intercept | 4.18 | 3.25, 5.12 | 8.85 | **<.001** | 0.08 | 2.45 (0.998) |
| Time | 0.09 | -0.13, 0.31 | 0.82 | 0.416 |  |  |
| **Associative model** |  |  |  |  |  |  |
| *BDI ~ positive expectations * time + (1 + positive expectations + time \| pID)* | | | | | | |
| Intercept | 27.63 | 24.38, 30.87 | 16.84 | **<.001** | 0.41 | 2.07 (0.672) |
| Positive expectations | -4.12 | -6.12, -2.12 | -4.08 | **0.004** |  |  |
| Time | -1.71 | -3.15, -0.27 | -2.36 | **0.020** |  |  |
| Positive expectations x time | -2.38 | -3.55, -1.21 | -4.04 | **<.001** |  |  |
| **Predictive model** |  |  |  |  |  |  |
| *BDI (t0-ti) ~ positive expectations (ti-1) * time + (1 \| pID)* | | | | | | |
| Intercept | 3.74 | 1.59, 5.89 | 3.46 | **<.001** | 0.07 | 2.27  (0.941) |
| Positive expectations | 2.14 | 0.51, 3.76 | 2.62 | **0.011** |  |  |
| Time | 1.82 | 0.52, 3.12 | 2.78 | **0.007** |  |  |
| Positive expectations x time | 1.07 | -0.22, 2.35 | 1.65 | **0.103** |  |  |
| **Translational models** |  |  |  |  |  |  |
| *BDI (t0-ti) * positive expectations (ti-1) ~ response criterion_happy>fearful_ (experiment) + (1 \| pID)* | | | | | | |
| Intercept | 0.20 | -0.06, 0.47 | 1.53 | 0.130 | 0.08 | 1.88 (0.245) |
| Response criterion _happy>fearful_ | 0.21 | 0.02, 0.41 | 2.21 | **0.032** |  |  |
| *BDI (t0-ti) * positive expectations (ti-1) ~ sensitivity_happy>fearful_ (experiment) + (1 \| pID)* | | | | | | |
| Intercept | 0.20 | -0.06, 0.46 | 1.51 | 0.135 | 0.18 | 1.91 (0.304) |
| Sensitivity _happy>fearful_ | 0.25 | 0.04, 0.45 | 2.45 | **0.019** |  |  |
|  |  |  |  |  |  |  |

^1^ first-order autoregressive correlation estimate. ^2^Durbin-Watson statistic and p-value

Testing two-sided for residual autocorrelation

**Table S7.** Model results with robustness diagnostics

|  | ß | 95% CI | t | P | W_e_  (Residual)^1^ | | W_ß_  (Random)^2^ | | |
| --- | --- | --- | --- | --- | --- | --- | --- | --- | --- |
|  |  |  |  |  | Down n/N (%)^3^ | Median  (Min, Max)^4^ | Down  n/N (%)^5^ | | Min, Max^6^ |
| **Trajectory models** |  |  |  |  |  | |  | | |
| *BDI ~ time + (1 + time \| pID)* | | | | | | | | | |
| Intercept | 31.47 | 28.72, 34.22 | 22.54 | **<.001** | 32/228  (14%) | 0.79  (0.22, 1.00) | 2/90  (2%) | 0.16, 1.00 | |
| Time | -1.48 | -2.32, -0.63 | -3.46 | **<.001** |  |  |  |  |  |
| *Positive expectations ~ time + (1 + time \| pID)* | | | | | | | | | |
| Intercept | 4.20 | 3.35, 5.05 | 9.73 | **<.001** | 30/178  (16.9%) | 0.70 (0.26, 0.98) | 2/90  (2.2%) | 0.26, 0.98 | |
| Time | 0.08 | -0.08, 0.24 | 1.04 | 0.297 |  |  |  |  |  |
| **Associative model** |  |  |  |  |  |  |  |  |  |
| *BDI ~ positive expectations * time + (1 + positive expectations + time \| pID)* | | | | | | | | | |
| Intercept | 27.98 | 24.56, 31.41 | 16.13 | **<.001** | 18/175  (10.3%) | 0.88  (0.48, 1.00) | 6/135  (4.4%) | 0.78, 1.00 | |
| Positive expectations | -3.66 | -6.18, -1.13 | -2.86 | **0.004** |  |  |  |  |  |
| Time | -1.54 | -3.78, 0.70 | -2.40 | 0.174 |  |  |  |  |  |
| Positive expectations x time | -2.15 | -3.35, -0.95 | -3.54 | **<.001** |  |  |  |  |  |
| **Predictive model** |  |  |  |  |  |  |  |  |  |
| *BDI (t0-ti) ~ positive expectations (ti-1) * time + (1 \| pID)* | | | | | | | | | |
| Intercept | 3.94 | 2.00, 5.69 | 4.47 | **<.001** | 21/134  (15.7%) | 0.66 (0.25, 1.00) | 9/45  (20.0%) | 0.40, 1.00 | |
| Positive expectations | 2.39 | 1.13, 3.64 | 3.75 | **<.001** |  |  |  |  |  |
| Time | 1.99 | 1.03, 2.96 | 4.08 | **<.001** |  |  |  |  |  |
| Positive expectations x time | 1.01 | 0.04, 1.97 | 1.85 | **0.038** |  |  |  |  |  |
| **Translational models** |  |  |  |  |  |  |  |  |  |
| *BDI (t0-ti) * positive expectations (ti-1) ~ response criterion_happy>fearful_ (experiment) + (1 \| pID)* | | | | | | | | | |
| Intercept | 0.15 | 0.02, 0.29 | 2.28 | **0.022** | 27/134 (20.2%) | 0.63 (0.22, 0.99) | 7/45  (15.6%) | 0.21, 1.00 | |
| Response criterion _happy>fearful_ | 0.10 | 0.00, 0.20 | 2.06 | **0.040** |  |  |  |  |  |
| *BDI (t0-ti) * positive expectations (ti-1) ~ sensitivity_happy>fearful_ (experiment) + (1 \| pID)* | | | | | | | | | |
| Intercept | 0.15 | 0.01, 0.29 | 2.07 | **0.038** | 27/134 (20.2%) | 0.63 (0.22, 0.99) | 7/45  (15.6%) | 0.20, 1.00 | |
| Sensitivity _happy>fearful_ | 0.12 | 0.01, 0.22 | 2.13 | **0.033** |  |  |  |  |  |
|  |  |  |  |  |  |  |  |  |  |

^1^Robustness weights applied to level-1 residuals. ^2^Number and proportion of residuals that were down weighted (weight < 1). ^3^Median and range of the residual robustness weights. ^4^Robustness weights applied to random-effects components (level -2). ^5^Number and proportion of random-effects components that were down weighted (weight < 1). ^6^Range of the random-effects robustness weights.

**Control for current treatment duration and depression history**

Predictive and translational models were re-run including treatment duration (time since admission minus time of first weekly assessment, in days) and overall illness duration (from first to most recent episode, in years) as control variables. None of the significant results in Table S3 were affected by these controls. Additionally, beta estimates for these duration parameters did not reach statistical significance in any analysis.

**Antidepressant dose change/switch**

To account for potential confounding by antidepressant changes, we included a binary time-varying variable (0 = no adjustment, 1 = adjustment) indicating dose changes or treatment switches (weekly dose changes of the primary antidepressant or class switches). As only nine participants switched antidepressants during the observation period, dose changes and switches were combined into a single variable. Including this indicator of medication stability in our predictive and translational models revealed no significant main or interaction effects, while the significance of the main effects persisted (Table S8).

**Table S8.** Model results for medication stability

|  | ß | 95% CI | t | P | R² (cond.) | R² (marg.) |
| --- | --- | --- | --- | --- | --- | --- |
| **Predicitve model** |  |  |  |  |  |  |
| *BDI (t0-ti) ~ positive expectations (ti-1) *(time + dose change/switch [yes/no]) + (1 \| pID)* | | | | | | |
| Intercept | 4.28 | 1.86, 6.71 | 3.49 | **<.001** | 0.619 | 0.064 |
| Positive expectations | 2.45 | 0.62, 4.28 | 2.65 | **0.009** |  |  |
| Dose change/switch | -0.36 | -2.99, 2.27 | -0.27 | 0.786 |  |  |
| Time | 2.03 | 0.45, 3.61 | 2.55 | **0.012** |  |  |
| Positive expectations x Dose change/switch | 2.01 | -1.00, 5.02 | 1.32 | 0.189 |  |  |
| Positive expectations x  Time | 1.70 | 0.11, 3.28 | 2.12 | **0.036** |  |  |
| **Translational Model** |  |  |  |  |  |  |
| *BDI (t0-ti) * positive expectations (ti-1) ~ response criterion_happy>fearful_ (experiment) * dose change/switch [yes/no]+ (1 \| pID)* | | | | | | |
| Intercept | 0.23 | -0.05, 0.51 | 1.60 | 0.111 | 0.527 | 0.078 |
| Response criterion | 0.23 | 0.03, 0.43 | 2.33 | **0.022** |  |  |
| Dose change/switch | -0.13 | -0.53, 0.26 | -0.68 | 0.495 |  |  |
| Response criterion x Dose change/switch | -0.04 | -0.43, 0.35 | -0.20 | 0.840 |  |  |
| *BDI (t0-ti) * positive expectations (ti-1) ~ sensitivity_happy>fearful_ (experiment) * medication dose change/switch [yes/no] + (1 \| pID)* | | | | | | |
| Intercept | 0.22 | -0.06, 0.50 | 1.59 | 0.115 | 0.535 | 0.095 |
| Sensitivity | 0.28 | 0.07, 0.48 | 2.66 | **0.009** |  |  |
| Dose change/switch | -0.12 | -0.52, 0.28 | -0.60 | 0.548 |  |  |
| Sensitivity x Medication dose change/switch | -0.13 | -0.51, 0.24 | -0.71 | 0.477 |  |  |
