# Supplementary material for: Acute placebo responsiveness predicts longitudinal expectation effects in antidepressant treatment
Source: Transl Psychiatry. 2026 May 13;16:241. doi: 10.1038/s41398-026-04070-x (PMC13172539; doi:10.1038/s41398-026-04070-x)
Supplement: Supplementary file 2 — Supplement 2 [file 41398_2026_4070_MOESM2_ESM.docx]

**Supplement 2**

Clinical trial protocol (including detailed methods and statistical plan)

CONSORT Checklist

| **1 General information** |
| --- |
| - 1. **Title of the research project**   Placebo responses as predictors of clinical expectation effects in depression |
| - 1. **Head of the research project:** |
| - - 1. **Name, position and address of the scientist responsible for the implementation of the research project**   Prof. Dr. Stefanie Brassen, Research Group Leader  Institute of Systems Neuroscience, University Medical Centre Hamburg-Eppendorf  Martinistr. 52, 20256 Hamburg  Phone: +49-40-7410-54865, Email: sbrassen@uke.de |
| - - 1. **Scientific qualification of the person responsible for the implementation**   Prof. Dr. Stefanie Brassen is research group leader at the Institute of Systems Neuroscience. She has many years of experience in the implementation, implementation and evaluation of neurobehavioral studies, especially in the areas of cognitive and emotion research. |
| - 1. **Names and addresses of other participating medical/scientific staff, cooperation partners, external laboratories, etc.**   Prof. Dr. Christian Büchel, Director  Institute of Systems Neuroscience, University Medical Centre Hamburg-Eppendorf  Telefon: +49-40-7410-54726, E-Mail: [buechel@uke.de](mailto:buechel@uke.de)  PD Dr. Gregor Leicht  Department of Psychiatry and Psychotherapy, University Medical Centre Hamburg-Eppendorf |
| - 1. **If available: Name and address of the sponsor and the monitor**   German Research Foundation Kennedyallee 40 53175 Bonn Germany |
| - 1. **Participation of other centres outside Hamburg:** |
| - - 1. **Names and addresses of other participating centres (e.g. clinics, practices)**   Not applicable |
| - 1. **Financing:**   *(Information in non-interventional studies (NIS) or observational studies mandatory)* |
| - - 1. **Who pays for all costs incurred in connection with the research project?**   Own funds or sponsors of the institution  Commercial Sponsor  Non-Commercial Sponsor  Public funding from: Deutsche Forschungsgemeinschaft, DFG  Other funding: |
| - 1. **Conflicts of interest or institutional dependencies of the Principal Investigator**   There are no possible economic and other interests related to the research project (e.g. financial agreement with the sponsor that could influence the results of the study due to its value; payments of other types by the sponsor, such as grants for ongoing research or equipment, consultant fees; ownership rights to the product under investigation; etc.)  The following economic and/or other interests exist: |

| **2 Objectives and/or purpose of the current research project** |
| --- |
| - 1. **Short and generally understandable description of the primary and, if applicable, the secondary goals or the purpose of this project** *(maximum 1/4 DIN A4 page!)*   Our study examines the influence of positive expectations — a proposed mechanisms underlying the placebo effect — on the behavioral processing of emotional faces and mood. Using a crossover design, patients experiencing an active depressive episode requiring treatment participate in an experimental protocol followed by an observational phase.  In both the control and placebo conditions of the experiment, participants are tasked with classifying subtle emotional facial expressions after receiving a saline nasal spray. In the placebo condition, positive expectations are induced by informing participants that the nasal spray contains oxytocin, along with the assertion that oxytocin has a beneficial effect on mood and facial emotion processing. In reality, all participants receive an isotonic saline solution. Following the experiment, patients undergo weekly assessments of their expectations regarding antidepressant treatment and are evaluated for depressive symptoms using the BDI-II (Beck Depression Inventory- II), both during their clinical stay and three months post-treatment.  The study aims to address the following questions:  - To what extent do depressive patients benefit from an induction of positive expectations? Do they exhibit similar effects as healthy individuals—such as improved mood and a positivity bias in emotion classification?  - Can individual responses to experimental expectation manipulation predict long-term effects of expectations on the clinical course of depression? |
| - 1. **Short and generally understandable summary** *(maximum 1/2 DIN A4 page!)* **the current state of knowledge (including information on previous benefits and risks, if applicable)**   Expectation effects in pain research, in the sense of placebo analgesia, have been well studied, while positive expectation effects on emotional processing and mood, such as those used to underpin antidepressant placebo effects, have been relatively little studied. In our previous work in healthy individuals, we could show that verbally induced positive expectations using a sham oxytocin treatment can induce a positivity bias in emotional processing in terms of lowering the threshold to detect happiness in subtle emotional face stimuli. It is unclear yet, if similar effects can be observed in patients with depression who often demonstrate a negativity bias that is related to symptoms and negative prognosis of the disease. Increased positivity has been observed following antidepressant treatment but given high placebo rates it could be speculated that this effect is at least partly mediated by positive treatment expectations alone. Moreover, it is also unclear yet whether experimental placebo effects in depression translate to real antidepressant expectation effects. Thus, the main goal of this combined experimental-observational study is to evaluate positive expectation effects on emotional processing in depressed inpatients and to investigate the predictive value of such placebo responsiveness in the experiment for longitudinal antidepressant expectation effects. Findings on this can have strong clinical implications with regard to the individual maximization of treatment effects (treatment + expectation). |

| **3 Planning and implementation of the study** |
| --- |
| - 1. **Planned study period (start / end)**   Spring 2021 – Spring 2025 |
| - 1. **Primary and, if applicable, secondary outcomes (clinically relevant parameters)**   Cross-over experiment: mood rating, response criterion and sensitivity (intercept/slope) of psychometric functions representing face classification  Longitudinal observational part: depressive symptoms (assessed via Beck’s Depression Inventory, BDI-II) and positive antidepressant treatment expectations |
| - 1. **Type of study design or research project** *(please tick all that apply)*   monocentric  multicentric  interventional study design  Study-related intervention (e.g. blood draw, additional X-ray examination, etc.)  non-interventional  retrospective  prospective  unblinded  singleblind  double-blind  Randomized  not randomized  cross-over  Observational study  Cross-sectional study  Cohort Study  Case-control study  pharmacokinetic examination  Research on biomaterials  with the establishment of a biobank  without setting up a biobank  Other Shape: |
| - 1. **Selection of study participants:** |
| - - 1. **Recruitment procedures (e.g. notice, advertisement, own database)**   *Note: For reasons of readability, the masculine form has been chosen in the text, but the information refers to members of both sexes.*  Patients are recruited through the Psychiatric Clinic of the University Medical Center Hamburg-Eppendorf (collaboration partner: Dr. Gregor Leicht). Patients will be contacted by the physicians and group announcements |
| - - 1. **Inclusion and exclusion criteria** *(please refer only to the relevant point in the study protocol)*   see study protocol, point 2.3.1 |
| - 1. **Information of designated study participants: place, time and information procedure (incl. reflection period), person providing information**   Location: UKE, Institute of Systems Neuroscience  Timing: Before the start of the study  Persons providing information: Principal investigator or participating scientific staff  Procedure: Potential participants are informed verbally and in writing about the course of study and its background. This is done based on Information Sheet A (Annex 01). Potential participants have the opportunity to ask questions at any time. After receiving this information, they are given sufficient time to consider their consent. Following the final examination, participants are informed that they have received placebo sprays without an active pharmaceutical ingredient. At this point, they are again given the opportunity to refuse further processing of their data. |
| - 1. **Measures / investigations:** |
| - - 1. **Which of the measures/examinations and equipment used in the study protocol correspond to clinical routine and which are carried out/used due to the study?**   All procedures belonging to the examination are study-related. |
| - 1. **Description of measures to minimise/avoid bias, including randomisation, blinding**   The order of the study days (d1, d2) is counterbalanced, i.e. 50% of the subjects receive the spray declared as oxytocin ("placebo") on day 1. The assignment of the subjects to these sequences is done by randomization (Matlab). The stimulus sequence in the paradigms is pseudo-randomized (specifications concern limited repetitions, stimulus conditions) |
| - 1. **Expected duration for the individual study participant**   For the screening (d0) 2h and for the two experimental measurement days 2h each. The weekly follow-up survey of approx. 15 minutes is carried out for the duration of the hospital stay |
| - 1. **Discontinuation and/or interruption of studies:** |
| - - 1. **Description of qualitative and quantitative discontinuation criteria and/or interruption rules for the overall study and the individual participants**   Individual participants: In accordance with the Declaration of Helsinki (supplemented in Tokyo, Venice, Hong Kong, Somerset West, Edinburgh), each individual participant may withdraw their participation at any time and without providing a reason. The examiner or physician is not allowed to exert undue influence on the participant to continue the study. However, the investigator will make reasonable efforts to find out the reasons for withdrawal while fully respecting the rights of the participant. The primary reason for termination will be recorded in the study documentation. The examining physician orders the immediate termination of the examination if a medical hazard of any kind is detected. If a possible danger to the participant arises from technical issues with the equipment used, the examiner orders immediate cessation of the study. Furthermore, the investigation must be terminated if the participant does not cooperate adequately in any other way and if the protocol is violated in a significant way.  Entire experiment: Stopping an experiment as a whole is the responsibility of the study leader. An experiment is initially stopped by the principal investigator at any time in the event of unacceptable safety risks to study participants (defined as a serious adverse event). This is followed by a consultation with the responsible ethics committee on the continuation and/or possible modification of the study. Other reasons for stopping an experiment are significant violations of the protocol and insufficient documentation. |

| - - 1. **Care of patients after premature discontinuation of studies (incl. statement on the further treatment of patients)**   Dropping out of studies has no consequences for the participants. There will be a final discussion with the study doctor, especially with regard to potential, but highly unlikely affective reactions. Discontinuation has no effect on the treatment of patients. Since they are usually already in psychiatric treatment, they can continue their treatment without consequences. |
| --- |
| - 1. **Provision of randomization codes and procedures for unblinding**   The placebo deception is clarified after the experiment. The subjects then have the opportunity to ask questions and the participants is given the opportunity to withdraw their data due to the deception. In the event of a withdrawal of the data, all data of the test subject will be deleted. |
| - - **List of case report forms and questionnaires used for study participants as well as information on their validation** - „Montgomery-Asberg Depressions Rating Skala“ (MADRS), Montgomery SA (1989), Perimed Fachbuch-Verlag-ges. - Beck Depression Inventory-II (BDI-II), Beck AT, Steer RA, Brown GK (1996), San Antonio, TX: The Psychological Corporation. - „Strukturiertes Klinisches Interview für DSM-5-Störungen“ (SCID-5) <https://www.testzentrale.de/shop/strukturiertes-klinisches-interview-fuer-dsm-5r-stoerungen-klinische-version.html> - Generic rating scale for previous treatment experiences, treatment expectations, and treatment effects (GEEE), Rief W, Nestoriuc Y, Mueller EM, Herrmann C, Schmidt K, Bingel U, PsychArchives. doi:10.23668/PSYCHARCHIVES.4717 |

| **4 Assessment of benefits and risks** |
| --- |
| - 1. **Justification of the need for human experiments on patients and/or healthy volunteers**   The planned series of experiments will be carried out to investigate the positive modulation of affective processes and mood by expectation. Therefore, these studies are only possible in humans. The inclusion of a group of patients with depressive episode allows us to test our assumptions about the importance of emotion processing for expectation effects, which play an important role in depression treatment. The methods used are suitable for human testing. |
| - 1. **Benefits / Advantages:** |
| - - 1. **For medicine and/or research**   A better understanding of the factors that modulate a positive effect of expectation on emotion processing and mood can improve the effect of a (pharmacological, e.g. antidepressant) therapy in the long term by optimal use of expectation effects. However, this experiment has no immediate benefit for medicine. |
| - - 1. **For the individual study participant**   There is no medical benefit for the individual study participant. By engaging with the study and the study design and background, the subject gains insight into current research. |
| - 1. **Risks / Disadvantages:** |
| - - 1. **Addressing possible risks and burdens related to the study participant**   There are no known side effects of the applied paradigm / saline application. |
| - - 1. **Safety Assessment Parameters**   During the experiment, the experimenter sits in the same room as the participant and can be contacted at any time. |
| - - 1. **Risk control measures**   All potential participants will be checked with regard to the exclusion criteria. Potential participants will be informed about the inclusion and exclusion criteria at an early stage and again in writing before final inclusion in the study. |
| - - 1. **Type and duration of follow-up of study participants after the occurrence of adverse events**   If adverse events occur, the subjects are followed up until the time of improvement. The study staff conducting the study as well as medical staff will provide help if necessary. Follow-up can be carried out at the study center, as well as in the adjacent emergency care at the University Hospital. |
| - - 1. **What study-related insurance has been taken out?**   Commuting accident insurance  Subject insurance for non-insurable studies  Other: The general liability principles apply, whereby a claim only arises in the event of culpable action by the employees of the UKE. The UKE has a liability insurance for these cases.  None |
| - - 1. **Measures in the event of a change in the risk situation (e.g. re-informing the study participants about adverse changes in the risk situation, adjustment of the discontinuation criteria, submission of correspondingly revised documents to the EC, etc.)**   At any point in the investigation, especially in the event of changes in the risk situation, the examiner weighs up the interests. In case of adverse development, the examination is not carried out. While the investigation is ongoing, the necessity of stopping the investigation will be examined. Any changes to the audit protocol during the course of the study will be immediately communicated to the Ethics Committee. Likewise, the Ethics Committee will be informed immediately of any serious events that may occur in the course of study. |

| - 1. **Vulnerable groups (e.g. minors, people who are unable to give consent, pregnant women, etc.)** |
| --- |
| - - 1. **Reason why the inclusion of vulnerable groups cannot be dispensed with**   Not applicable |
| - - 1. **In the case of the inclusion of persons who may not be able to give consent (e.g. emergency): measures to determine the capacity to consent**   Not applicable |

| **5 Expense allowance / allowances for study participants** |
| --- |
| - 1. **Remuneration/reimbursement of costs as well as material resources and other benefits**   Behavioral measurements/questionnaires are remunerated at 15€/h. |

| **6. Statistics** |
| --- |
| - 1. Was a biometrician involved in the planning?   No  Yes -> Name and address: |
| - 1. **Planned number of cases or number of samples**   N = 63 participants are planned including 30% drop-out. |
| - 1. **Justification of the number of cases**   The calculated sample size for all study components is deemed sufficient to detect at least medium effect sizes (d = 0.50). Assuming an alpha level of 5% and a power of 90%, a minimum of 44 participants is required for the experimental part, which involves repeated measures within factors (G*Power 3.1). For the multilevel models, power analyses conducted using the `simr` package in R, based on 500 simulation runs, indicates that a sample of at least 40 participants would provide a power greater than 90% (93.0%, 95% CI: 90.4%–95.1%) to detect a medium effect size (d = 0.50) on fixed effects, assuming an average of 4 repeated measurements per participant. The initial recruitment strategy accounts for an estimated 30% dropout rate, given the expectation that not all patients will participate in the longitudinal assessments.  Literature:  Confirmatory study design -> statistical justification incl. indication of hypotheses, target parameters, expected effects, drop-out rate, subgroups if applicable, significance (power), significance level:  Other: |
| - 1. **Aim of the evaluation**   Generation of hypotheses (exploratory)  Testing of hypotheses (confirmatory)  Superiority test  Equivalence check  Non-inferiority test  is omitted due to the design of the research project (e.g. register, etc.) |
| - 1. **Planned statistical analysis methods, including the dates for any planned interim evaluations.**   Data will be processed and analyzed using MATLAB (Mathworks, MA) and R / RStudio (R v4.3.2). Statistical analyses employ the general linear model framework, including repeated measures ANOVA (rmANOVA), one-sample t-tests, Pearson correlation, and multilevel models (LMM). Significance is set at α=0.05 (two-sided).  EC-Task data analyses follow previous protocols (Baker et al., SciRep 2022). Classification accuracy at each intensity and emotion in the control condition serves as baseline; responses in the placebo condition will be regressed onto control within participants, yielding intercepts and slopes to assess response criterion and sensitivity changes.  Longitudinal data will be analyzed with LMMs (lme4), initially with random intercepts and slopes. Model comparisons use likelihood ratio tests, AIC, and BIC; if adding slopes does not significantly improve fit, results from simpler models with only intercepts are reported, balancing complexity and fit. Interim evaluations are carried out after the individual samples have been completely collected. |

| **7 Data flow, data protection concept, archiving** |
| --- |
| - 1. **Description of the data flow from collection, transfer to archiving, taking into account all involved bodies and functionaries (e.g. clinic, sponsor, other external bodies, etc.)**   The personal data collected in the course of the study after the study participant's declaration of consent, are subject to confidentiality and the provisions of data protection law. They are recorded in paper form and on data carriers at the Institute for Systems Neuroscience of the University Medical Center Hamburg – Eppendorf and stored pseudonymized (encrypted) for a maximum period of 10 years. In pseudonymization (encryption), the name and other identification features (e.g. parts of the date of birth) are replaced by e.g. a multi-digit combination of letters or numbers, also known as a code, in order to exclude or significantly complicate the identification of the study participant. Access to the "key", which enables a personal assignment of the study participant's data, is only available to the study leader. The evaluation and use of the data by the head of the study and potential collaborators is carried out in pseudonymized form. The data collected in the context of the study will only be passed on in anonymized form. The same applies to the publication of the study results.  **Note on the privacy sections used**  The data protection sections used in the present application have been formulated together with Dr. Menzel and Mr. Jaster, responsible for science at the Hamburg Commissioner for Data Protection and Freedom of Information. The relevant documents have been submitted to the Ethics Committee. |

| **8 Biomaterials** |
| --- |
| - 1. **Purpose of sampling**   Not applicable  Establishment of a new biobank (use not only for a very specific research project)  Supplementing an existing biobank for unspecified purposes; Edited by the EK Hamburg:  Sample collection for specific study purposes:  genetic tests  Other Investigations |
| - 1. **Sample Source**   Not applicable  Residual material from clinical routine  Invasive removal of additional material during an intervention that is already planned  Invasive sample collection as part of an additional procedure  Withdrawal from an existing biobank  Other: |
| - 1. **Type of biomaterial**   Not applicable  Blood  Cells  Fabric  DNA  Other: |
| - 1. **Description of the sample transfer (if necessary with the attachment of a diagram) from collection, transfer to storage and destruction, taking into account all involved bodies and officials (e.g. clinic, sponsor, other external bodies, etc.)**   Not applicable |
| - 1. **Presentation of the data protection concept from removal to destruction, taking into account the above-mentioned bodies/functionaries in compliance with the EC data protection model or the model clarification of the Ethics Committees Working Group**   Not applicable |
| - 1. **Duration of storage and time of anonymisation or destruction; if applicable, including a description of how to deal with residual material**   Not applicable |

1. **Personal information**

**Names and offices of the Head of Clinical Trials (LPK) (corresponding to § 40 AMG or § 20 MPG) and his staff**

| Prof. Dr. Stefanie Brassen, Research Group Leader  Institute of Systems Neuroscience (ISN), University Medical Centre Hamburg-Eppendorf (UKE)  Martinistr. 52, 20246 Hamburg  Phone: +49-40-7410-54865, E-mail: [sbrassen@uke.de](mailto:sbrassen@uke.de) |  |
| --- | --- |
| Prof. Dr. med. Christian Büchel, Director  ISN, UKE  Telefon: 040-7410-54726, E-Mail: [buechel@uke.de](mailto:buechel@uke.de) | |

Eun Jin Shim, medical student, ISN, UKE

Leonie Schmidt, MSc, ISN, UKE

Jonas Rauh, MD, ISN, UKE

- 1. **Proof of the scientific qualification of the person responsible for the implementation**

Prof. Dr. Stefanie Brassen is a psychologist with a habilitation and doctorate and is a research group leader at the Institute of Systems Neuroscience. She has many years of experience in the implementation and evaluation of neuroscientific imaging and behavioral studies, especially in the areas of cognitive and emotion research.

- 1. **Financing of the study**

The study is part of the Transregio SFB 289 ("Treatment expectation", collaboration research centre of the Universities of Hamburg, Marburg and Essen) and funded by the German Research Foundation (Project-ID 422744262).

1. **Description and scientific justification of the project**
   1. **Objectives**

Our study examines the influence of positive expectations — a proposed basis of the placebo effect — on the behavioral processing of emotional faces and mood. Utilizing a cross-over design, patients experiencing an active depressive episode requiring treatment participate in an experimental protocol followed by an observational phase.

In both the control and placebo conditions of the experiment, participants are tasked with classifying subtle emotional facial expressions after receiving a saline nasal spray. In the placebo condition, positive expectations are induced by informing participants that the nasal spray contains oxytocin, along with the assertion that oxytocin has a beneficial effect on mood and facial emotion processing. In fact, all participants receive an isotonic saline solution. Following the experiment, patients undergo weekly assessments of their expectations regarding antidepressant treatment and are evaluated for depressive symptoms using the Beck’s Depression inventory (BDI-II), both during their clinical stay and three months after discharge.

The study aims to address the following questions:

- To what extent do depressive patients benefit from an induction of positive expectations? Do they exhibit similar effects as healthy individuals—such as improved mood and a positivity bias in emotion classification?

- Can individual responses to experimental expectation manipulation predict long-term effects of expectations on the clinical course of depression?

- 1. **Presentation of the current state of knowledge**

In our preliminary work, we were able to show that expectation effects on emotional processing induced by verbal instructions enhance mood and evoke a positivity bias in emotional processing in healthy participants (Baker et al., 2022, Rauh et al., 2023). The finding of the placebo-induced positivity effect is particularly interesting in that negativity bias is often observed in depressed patients (Gollan et al., 2010). In predictive coding theory, these are attributed to a reduced effect of positive prediction errors and an overprecise representation of negative expectations (Kube et al., 2020). The treatment of depression could therefore be understood as equipping the brain with the means to correct its internal model (i.e. its predictions) of the world by changing relevant statistical structures, and thus to become "less pessimistic". Treatment with antidepressants has been shown to reduce negativity bias while improving mood (Harmer et al., 2009). Our results so far indicate the possibility that this could be at least partly due to expectation effects. Our study will investigate whether negativity bias in depression can be reduced by inducing positive expectations, and to what extent this is modulated by previous experience (Anderson & Stebbins, 2020). In addition, we would like to examine whether the individual response to experimental-induced expectations can be used to predict the long-term modulation of clinical effects by treatment expectations. Findings on this can have strong clinical implications with regard to the individual maximization of treatment effects (treatment + expectation)

- 1. **Study Design and Duration**
     1. Participants

For this study, N = 63 inpatients with a diagnosis of major depressive episode (MDD) will be recruited by the treating physicians via the Clinic and Polyclinic for Psychiatry and Psychotherapy of the University Medical Center Hamburg-Eppendorf (cooperation partner: Senior Consultat Dr. Gregor Leicht). Psychiatric diagnoses including comorbidities are verified by means of a structured interview (SCID-5, <https://www.testzentrale.de/shop/strukturiertes-klinisches-interview-fuer-dsm-5r-stoerungen-klinische-version.html>). In addition, depressive symptoms will be quantified using a standardized clinical interview based on the Montgomery-Asberg Depression Rating Scale (MADRS) and the Beck Depression Inventory II (BDI-II). The patients are examined by a doctor with psychiatric training. Any antidepressant or other medication is protocolled. For all studies, the planned groups consist of 50% women. All participants must declare their participation in the study in writing.

**Inclusion Criteria**

- Subjects of legal age who are fully capable of contracting and enlightening
- Age: 18 – 60 years
- Consent to the examination
- Primary diagnosis of MDD according to DSM-5
- For the longitudinal observational part only: ongoing treatment with antidepressants

**Exclusion criteria**

- Severe internal diseases (e.g. untreated hyperthyroidism and hypothyroidism, liver or kidney diseases)
- Participation in other drug-associated studies
- Current or previous neurological diseases
- Psychotic disorders
- Substance use disorders
- Acute suicidality
  - 1. Sample sizes

The calculated sample size for all study components is deemed sufficient to detect at least medium effect sizes (d = 0.50). Assuming an alpha level of 5% and a power of 90%, a minimum of 44 participants is required for the experimental part, which involves repeated measures within factors (G*Power 3.1). For the multilevel models, power analyses conducted using the `simr` package in R, based on 500 simulation runs, indicates that a sample of at least 40 participants would provide a power greater than 90% (93.0%, 95% CI: 90.4%–95.1%) to detect a medium effect size (d = 0.50) on fixed effects, assuming an average of 4 repeated measurements per participant. The initial recruitment strategy accounts for an estimated 30% dropout rate, given the expectation that not all patients will participate in the longitudinal assessments.

- - 1. General procedure

The experimental study part uses a controlled, randomized, cross-over design. For each participant there is a screening day (d0) and 2 experimental days (d1, d2). On the screening day (duration: ~ 2h), the examination involves a clinical interview, quantification of depression severity using the Montgomery-Åsberg Depression Rating Scale (MADRS), and a baseline (t0) assessment of our primary outcome for depressive symptoms with the Beck Depression Inventory-II (BDI-II).

The two experimental days (each approximately 2 hours long) are scheduled to take place at intervals of about one week. During these sessions, participants undergo the study experiment combined with either intranasal "placebo" or "oxytocin” administration (both containing isotonic saline solution) in a counterbalanced order. Initially, participants' current mood is assessed, and they complete a brief training of the paradigm (see below). Subsequently, their expectation of positive effects from intranasal oxytocin on mood and affective processing is actively induced via a self-developed video documentation, enabling a highly standardized experimental induction of expectation. After this presentation, participants administer four puffs of the nasal spray—two in each nostril—with the pretext of a lottery revealing whether it contains oxytocin (placebo session) or saline solution (control session). In both cases, only the isotonic saline solution is actually administered. Following an assessment of their expectations regarding potential mood changes caused by the nasal spray, a second training session (Training+) is initiated. Afterwards, participants perform the paradigm (see below). Following the second experimental day, enrolled participants of the longitudinal part will be assessed weekly until discharge on their depressive symptoms (BDI-II), current antidepressant treatment and related treatment expectations and experiences. The observational assessment will be repeated 12 weeks after the experiment via telephone. After their last assessment, participants are debriefed and informed that they received placebo sprays without active medication. At this point, they are given the opportunity to decline further use or processing of their data.

- - 1. Behavioral measurements, recording of previous experiences and treatment courses

The current mood is measured using a visual analogue scale (VAS, 0-100). Depression symptoms are recorded with the BDI-II. Treatment expectations and experiences during the experiment and the longitudinal part are recorded by means of adapted GEEE questionnaires (Rief et al., 2021).

- - 1. Video documentation about oxytocin and application

To induce the expectation of a positive effect of intranasal oxytocin on the perception of positive emotions and mood state, participants will watch a self-developed 5-minute video documentary in which an "expert" explains how oxytocin can modulate the perception of emotional information (e.g., emotional faces). All material presented in this video is based on published research. The video concludes that a nasal spray with oxytocin can i) enhance the perception and effect of positive facial expressions, ii) reduce the perception and effect of negative facial expressions, and iii) improve the mood of participants. The video will be shown on both scan days. The participants then receive a nasal spray with isotonic saline solution. In a counterbalanced design with randomized allocation, they then receive the corresponding information on one day that it is "oxytocin" while on the other day the information "saline solution" is conveyed by the experimenter. This approach corresponds to that of our previous studies (Baker et al., 2022; Mostauli et al., 2025).

- - 1. Paradigm
       1. *Emotion classification task*

In this task, participants are presented with facial expressions displaying varying levels of emotional intensity, and are asked to detect the (subtle) emotion being expressed (happy, fearful, or neutral). Based on an extensive validation procedure (Baker et al., 2022), images are selected for each emotion that result in classification accuracies of 25%, 37.5%, 50%, and 62.5%. Additionally, neutral faces of each identity are shown. There are two stimulus sets, each containing 352 images (8 identities, 4 women, five intensity levels, 4 representations per image), which are balanced in terms of difficulty and are used interchangeably across the two test days. Each trial begins with a 1-second fixation cross, followed by the presentation of the face for 1.5 seconds, then a selection screen, and a jittered inter-trial interval of 2 to 3.5 seconds. During the Training+ session in the placebo condition, a manipulated training is conducted in which a subtle, hidden increase in the intensity of happy faces makes them easier to recognize, thus creating a positive "treatment" experience (Baker et al., 2022).

- - 1. Statistical analyses

Data are processed and analyzed using MATLAB (Mathworks, MA) and R / RStudio (R v4.3.2). Statistical analyses employ the general linear model framework, including repeated measures ANOVA (rmANOVA), one-sample t-tests, Pearson correlation, and multilevel models (LMM). Significance is set at α=0.05 (two-sided).

For the analysis of the behavioral data of the emotion classification task, the detection rates for the individual emotions between placebo and control conditions are compared using psychometric functions. This makes it possible to separate expectation effects on response tendencies (intercept) and ability to discriminate (slope). This procedure corresponds to the analysis of the preliminary work from our laboratory (Baker et al., 2022).

Longitudinal data including depressive symptoms (BDI), treatment expectation, and finally the inclusion of experimental responsiveness are analyzed with LMMs, initially with random intercepts and slopes. Model comparisons use likelihood ratio tests, AIC, and BIC; if adding slopes do not significantly improve fit, results from simpler models with only intercepts are reported, balancing complexity and fit.

**2.4 Hypotheses**

- Alleged “oxytocin” treatment enhances mood (in the VAS) and induces a positivity bias in emotional processing (i.e., lowering the threshold to detect happiness in subtle facial stimuli)
- Expectations of antidepressant efficacy predict decline in depressive symptoms during the clinical stay
- Individual placebo responsiveness in the experiment predicts clinical expectation-outcome associations, i.e. the higher the responsiveness in the cross-over experiment the stronger the relationship between clinical expectations and outcome

1. **Bibliography**

Baker, J., Gamer, M., Rauh, J., & Brassen, S. (2022). Placebo induced expectations of mood enhancement generate a positivity effect in emotional processing. *Scientific Reports*, *12*(1), 5345. https://doi.org/10.1038/s41598-022-09342-2

Gamer, M., & Büchel, C. (2009). Amygdala Activation Predicts Gaze toward Fearful Eyes. *Journal of Neuroscience*, *29*(28), 9123–9126. https://doi.org/10.1523/JNEUROSCI.1883-09.2009

Gamer, M., Zurowski, B., & Büchel, C. (2010). Different amygdala subregions mediate valence-related and attentional effects of oxytocin in humans. *Proceedings of the National Academy of Sciences of the United States of America*, *107*(20), 9400–9405. https://doi.org/10.1073/pnas.1000985107

Gollan, J. K., McCloskey, M., Hoxha, D., & Coccaro, E. F. (2010). How do depressed and healthy adults interpret nuanced facial expressions? *Journal of Abnormal Psychology*, *119*(4), 804–810. https://doi.org/10.1037/a0020234

Kube, T., Schwarting, R., Rozenkrantz, L., Glombiewski, J. A., & Rief, W. (2020). Distorted Cognitive Processes in Major Depression: A Predictive Processing Perspective. *Biological Psychiatry*, *87*(5), 388–398. https://doi.org/10.1016/j.biopsych.2019.07.017

Mostauli, A., Rauh, J., Gamer, M., Büchel, C., Rief, W., & Brassen, S. (2025). Placebo treatment entails resource-dependent downregulation of negative inputs. *Scientific Reports*, *15*(1), 9088. https://doi.org/10.1038/s41598-025-93589-y

Rief, W., Nestoriuc, Y., Mueller, E. M., Hermann, C., Schmidt, K., & Bingel, U. (2021). *Generic rating scale for previous treatment experiences, treatment expectations, and treatment effects (GEEE)*. https://www.psycharchives.org/en/item/a0bc93a2-9e03-4bba-97b0-8f478b2a39ca

1. **List of abbreviations**

ANOVA - Analysis of variance

BDI-II - Beck Depression Inventory II

DFG - Deutsche Forschungsgemeinschaft

GEEE - Generic rating scale for previous treatment experiences, treatment

expectations, and treatment effects

MADRS - Montgomery-Asberg Depression Rating Scale

MDD - Major Depressive Disorder

VAS - Visual Analog Scale

| 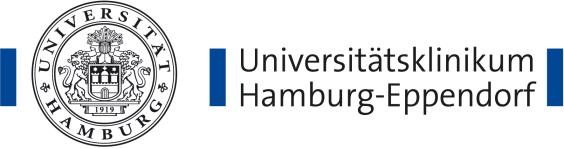 | **Institut für Systemische**  **Neurowissenschaften**  Prof. Christian Büchel  Direktor |  | **Zentrum für Experimentelle Medizin**   Martinistraße 52  20246 Hamburg  Telefon: (040) 7410-54865  Telefax: (040) 7410-59955  sbrassen@uke.de  [www.uke.de](http://www.uke.de)  Ansprechpartner/-in  Prof Dr. Stefanie Brassen |
| --- | --- | --- | --- |
| **Bogen A**   - Merkblatt zur Aufklärung und Einwilligungserklärung -   Studie: „Oxytocinwirkung auf die Verarbeitung während einer  kognitiven Aufgabe“ | | | |

Name, Vorname: .............................................................................

Adresse: ..........................................................................................

Geburtsdatum: ................................................................................

Telefon: ...........................................................................................

E-Mail: .............................................................................................

Sehr geehrte Interessentin/Patientin,

sehr geehrter Interessent/Patient,

wir möchten Sie herzlich einladen, an der Studie ”Oxytocinwirkung auf die Verarbeitung während einer kognitiven Aufgabe” teilzunehmen. In dieser Studie soll der Einfluss des Hormons Oxytocin auf kognitive Prozesse erforscht werden. Dazu finden zwei Messungen statt. Im weiteren Verlauf möchten wir untersuchen, wie die Reaktion in der kognitiven Aufgabe mit dem weiteren Behandlungsverlauf zusammenhängt. Die Teilnahme ist freiwillig und Sie können jederzeit und ohne Angabe von Gründen Ihre Teilnahme widerrufen, ohne dass Ihnen daraus Nachteile entstehen. Wenn Sie Fragen haben, wenden Sie sich an unsere Mitarbeiter, wir geben Ihnen gerne Auskunft.

**Ziel der Studie**

Bisherige Untersuchungen haben gezeigt, dass das körpereigene Hormon Oxytocin die Verarbeitung von sozial relevanten Reizen wie Gesichtern verbessern kann. Wie dies im Detail funktioniert, ist momentan noch nicht ausreichend geklärt. In dieser Studie sollen die kognitiven Prozesse, die an diesem Mechanismus beteiligt sind, genauer untersucht werden.

**Ablauf der Studie**

***Screening***: Vor dem eigentlichen Experiment laden wir Sie zu einer Vorbesprechung ein. Diese wird in der psychiatrischen Klinik der Universitätsklinikum Eppendorf stattfinden. Hier werden wir das Experiment und den Ablauf der Studie besprechen. Währenddessen haben Sie außerdem selbst die Zeit und Möglichkeit, Fragen zu stellen. Sie haben außerdem die Möglichkeit bei einem Zusatzexperiment teilzunehmen, bei dem Sie Speichelproben abgeben müssen. Bei Interesse erhalten Sie hierzu gesonderte Informationen. Die Teilnahme dabei ist optional.

Wenn Ihre Einverständniserklärung vorliegt, wird ein neuropsychologischer Test zur Informationsverarbeitung am Computer durchgeführt. Des Weiteren werden Ihnen Fragebögen zur Erfassung der Stimmung und bestimmter Persönlichkeitseigenschaften vorgelegt. Außerdem wird eine Untersucherin oder ein Untersucher einige Fragen zu aktuellen Beschwerden stellen. Diese Untersuchungen dauern mit Pause maximal **1,5-2 Stunden**.

***Studientag 1 & 2***: Studientag 1 und 2 sind vom Ablauf identisch und sollten einige Tage auseinander liegen. Nach Ihrer Ankunft im Labor erfassen wir zunächst Ihre aktuelle Stimmung. Im Anschluss sehen Sie ein kurzes Video, in dem Ihnen ein Experte die Wirkweise von Oxytocin erklärt. Im Anschluss wird ausgelost, ob sie an dem jeweiligen Studientag Oxytocin oder eine Kochsalzlösung („Kontrollbedingung“) als Nasenspray erhalten. Anschließend wird Ihnen das entsprechende Nasenspray verabreicht (2 Sprühstöße pro Nasenöffnung). Um verdeckte Effekte durch Erwartungen auszuschließen, ist die Gabe nicht verblindet, d.h. Sie erfahren an jedem Tag, ob Sie Oxytocin oder Kochsalz erhalten. Sie führen dann zwei Aufgaben durch. Bei der ersten Aufgabe geht es um das Erkennen von Emotionen. Beide Aufgaben werden im Vorfeld ausführlich mit Ihnen trainiert. Sie haben dabei jederzeit die Möglichkeit Fragen vor Ort zu stellen. Zum Abschluss finden noch einige Befragungen statt. Die gesamte Verweildauer beträgt pro Studientag etwa **1 Stunde**.

***Wöchentliche Befragung*:** Sie erhalten von uns in wöchentlichen Abständen einige Fragebögen bezüglich Ihrer aktuellen Befindlichkeit. Für die Fragebögen werden Sie voraussichtlich etwa **30 Minuten** pro Woche benötigen. Diese Fragebögen werden Sie für die Dauer Ihres stationären Aufenthaltes erhalten.

***Vergütung***

Für die Untersuchungen am Screening-Tag erhalten Sie 12 Euro / Stunde. Für die Messungen an den Studientagen 1 und 2 erhalten Sie 15 Euro / Stunde. Pro wöchentlicher Befragung erhalten Sie 6 Euro. Bei Abschluss werden Ihnen demnach für das gesamte Experiment ca. 90 Euro auf ein deutsches Bankkonto überwiesen.

**Gibt es irgendwelche möglichen Risiken für mich, wenn ich an der Studie teilnehmen werde?**

***Oxytocin***

Oxytocin ist ein körpereigenes Hormon, das soziales Verhalten positiv beeinflusst. In Studien konnte gezeigt werden, dass Oxytocin die Wahrnehmung positiver Reize sowie die Stimmung verbessern kann. Bei der in dieser Studie verabreichten Menge von Oxytocin sind aufgrund der Verabreichungsart als Nasenspray und der geringen Menge keine relevanten Nebenwirkungen, außer einem kurzzeitigen Kribbeln oder Brennen in der Nase, zu erwarten.

**Datenschutz**

Gemäß den datenschutzrechtlichen Bestimmungen benötigen wir Ihr Einverständnis zur Speicherung und Verwendung der von Ihnen im Rahmen der Studie erfassten Daten. Die Speicherung und Verarbeitung Ihrer personenbezogenen Daten erfolgt pseudonymisiert^^[[1]](#footnote-1)^^, d.h. in namentlich nicht kenntlicher Form. Dies bedeutet, dass die Daten nur mit einem Ihnen zugewiesenen Pseudonym verwendet werden, z.B. VP5 für Versuchsperson Nr. 5. Die Pseudonymisierung der Daten erfolgt durch den Studienleiter oder, im Urlaubs- oder Krankheitsfall, seinen Vertreter und ist nur diesem bekannt. Weder bei der Erhebung der Daten noch im Rahmen der Auswertung werden Ihr Name oder Ihre Initialen gespeichert. Um verbesserte Auswertungsprogramme nutzen zu können, werden die Messdaten u.U. in Zusammenarbeit mit Wissenschaftlern anderer Arbeitsgruppen ausgewertet, jedoch nur in pseudonymisierter^1^ oder anonymisierter Form, so dass andere Wissenschaftler ohne Ausnahme keine Kenntnis davon erhalten, zu welcher Person die analysierten Daten gehören. Eine Veröffentlichung von Studienergebnissen erfolgt anonymisiert^2^. Die Daten werden gespeichert, solange es der Studienzweck erfordert, spätestens jedoch nach zehn Jahren oder bei einem Widerruf der Einverständniserklärung zur Teilnahme an der Studie bzw. der Speicherung der Daten gelöscht.

Die folgende Tabelle soll Ihnen eine Übersicht über die im Rahmen dieser Studie erhobenen und gespeicherten Daten geben.

| *Art der Daten* | Studienspezifische Daten |
| --- | --- |
| *Kennbuchstabe des Bogens* | A |
| *Beispiele* | Reaktionszeiten im Experiment, Fragebögen Antworten |
| *Ort der Speicherung* | Institut für Systemische  Neurowissenschaften |
| *Pseudonymisierung^2^* | Ja |
| *Zugriff auf den Pseudonymisierungsschlüssel* | Prof. Dr. Stefanie Brassen  Studienleiterin |
| *Zugriff auf die Daten* | Studienmitarbeiter |
| *Verantwortliche/r (Stellvertreter/in)* | Prof. Dr. Stefanie Brassen (Prof. Dr. Christian Büchel) |

Ihr Einverständnis zur Speicherung der in dieser Studie produzierten Daten können Sie jederzeit widerrufen, ohne dass Ihnen daraus Nachteile entstehen. Außerdem können Sie unentgeltlich Auskunft über die gespeicherten Daten erhalten und deren Berichtigung oder Löschung verlangen. Die Studie berücksichtigt stets den Grundsatz, dass die Belange der Versuchsperson selbstverständlich Vorrang haben. Für Sie entstehen aufgrund der nicht-invasiven Methoden, bei Einhaltung der Sicherheitsbestimmungen, keinerlei Risiken; der mögliche Erkenntnisgewinn für die Forschung ist hingegen hoch. Die Zahl der Versuchsteilnehmer wird auf das unbedingt notwendige Maß beschränkt.

Den/die Verantwortlichen (Prof. Dr. Stefanie Brassen, Prof. Dr. Christian Büchel) für die Speicherung Ihrer Daten erreichen Sie unter

Institut für Systemische Neurowissenschaften

Universitätsklinikum Hamburg-Eppendorf, Geb. W34

Martinistraße 52

20246 Hamburg

oder telefonisch unter 040/7410-54865 bzw. per E-Mail über [sbrassen@uke.de](mailto:sbrassen@uke.de).

Bei Beschwerden richten Sie sich an einen der o.a. Verantwortlichen oder den Landesdatenschutzbeauftragten unter

Der Hamburgische Beauftragte für Datenschutz und Informationsfreiheit

Kurt-Schumacher-Allee 4

20097 Hamburg

oder telefonisch unter 040/428 54-4040, per Fax an 040/4279-11811,

per E-Mail an [mailbox@datenschutz.hamburg.de](mailto:mailbox@datenschutz.hamburg.de) oder im Internet unter www.datenschutz-hamburg.de.

**Angaben zum Versicherungsschutz:**

Es gelten die allgemeinen Haftungsgrundsätze, wobei sich ein Anspruch nur bei schuldhaftem Handeln der Mitarbeiter des UKE ergibt. Das UKE verfügt für diese Fälle über eine Haftpflichtversicherung. Diese leistet Ersatz für Personen- und Sachschaden, die Sie infolge Ihrer Teilnahme an der Studie erleiden, sofern ein schuldhaftes Verhalten des Klinikpersonals ursächlich ist.

**Allgemeine Hinweise:**

Diese Studie ist von der unabhängigen Ethikkommission der Ärztekammer Hamburg hinsichtlich ihrer medizinischen, rechtlichen und ethischen Vertretbarkeit beraten worden. Die Verantwortung für die Durchführung verbleibt jedoch beim Studienleiter.

Bitte beachten Sie, dass in manchen Studien die Studienteilnehmer aus wissenschaftlich-methodischen Gründen nicht vollumfänglich über alle Ziele und Inhalte der Untersuchung informiert werden. Dies kann beispielsweise bei bestimmten psychologischen Fragestellungen der Fall sein, bei dem unbewusste Vorgänge wie z.B. Lernmechanismen untersucht werden sollen. Ein solches Vorgehen geschieht immer in verantwortungsvoller und ethisch vertretbarer Weise und ausschließlich dann, wenn die wissenschaftliche Fragestellung nicht anders beantwortet werden kann. In keinem Fall erhalten Sie Medikamente / Substanzen über die Sie nicht im Rahmen der Aufklärung ausführlich informiert wurden und in deren Gabe Sie nicht ausdrücklich eingewilligt haben. Ebenso werden keinesfalls invasive Prozeduren (wie Schmerzreize, Blutentnahmen etc.) im Rahmen der Untersuchung durchgeführt, in die Sie nicht ausdrücklich eingewilligt haben. In jedem Fall informieren wir Sie nach Abschluss der Studie vollumfänglich über die Sie betreffenden Aspekte der Studie.

Diese Broschüre und auch eine Kopie der unterschriebenen Einwilligungserklärung sind für Ihre Unterlagen bestimmt. Bitte bewahren Sie diese auf.

**Einwilligungserklärung**

*Herr / Frau ........................................................................hat mich in einem Aufklärungsgespräch ausführlich über die Studie „Oxytocinwirkung auf die Verarbeitung während einer kognitiven Aufgabe“ informiert. Dabei konnte ich alle mir wichtig erscheinenden Fragen, insbesondere zu Risiken, Komplikationen und Nebenwirkungen der Studie, stellen.*

🞏 Ja 🞏 Nein

*Ich weiß, dass die Teilnahme freiwillig ist und ich die Studie jederzeit abbrechen kann ohne dass mir daraus Nachteile entstehen. Ich weiß, dass mir durch die Teilnahme an der Studie keine persönlichen Vorteile entstehen.*

🞏 Ja 🞏 Nein

*Ich bin mit der Speicherung der im Rahmen der Studie erhobenen Daten, wie oben beschrieben, einverstanden und habe mein Auskunfts- und Beschwerderecht zur Kenntnis genommen.*

🞏 Ja 🞏 Nein

*Ich habe keine weiteren Fragen, fühle mich ausreichend informiert und willige nach ausreichender Bedenkzeit mit meiner Unterschrift in die Teilnahme an der Studie ein.*

🞏 Ja 🞏 Nein

*Eine Kopie dieser Bögen sowie dieses Merkblatts wurde mir ausgehändigt.*

Datum: ........................................... Unterschrift: ......................................................................................

--------------------------------------------------- wird vom Mitarbeiter ausgefüllt ---------------------------------------------

Name der aufklärenden Person: ......................................................................................

Datum: ........................................... Unterschrift: ......................................................................................

1. Pseudonymisieren ist das Ersetzen des Namens und anderer Identifikationsmerkmale durch ein Kennzeichen zu dem Zweck, die Identifizierung des Betroffenen auszuschließen oder wesentlich zu erschweren (§3, Abs 6a Bundesdatenschutzgesetz)

   ^2^ Anonymisierung ist das Verändern personenbezogener Daten derart, dass die Einzelangaben über persönliche oder sachliche Verhältnisse nicht mehr oder nur mit unverhältnismäßig großem Aufwand an Zeit, Kosten und Arbeitskraft einer bestimmten oder bestimmbaren natürlichen Person zugeordnet werden können (§3, Abs. 6 Bundesdatenschutzgesetz) [↑](#footnote-ref-1)
